# Landscape level associations between birds, mosquitoes and microclimates: possible consequences for disease transmission?

**DOI:** 10.1186/s13071-024-06239-z

**Published:** 2024-03-26

**Authors:** Louie Krol, Laure Remmerswaal, Marvin Groen, Jordy G. van der Beek, Reina S. Sikkema, Martha Dellar, Peter M. van Bodegom, Gertjan W. Geerling, Maarten Schrama

**Affiliations:** 1 Institute of Environmental Sciences, Leiden University, Leiden, The Netherlands; 2 Deltares, Daltonlaan 600, Utrecht, The Netherlands; 3 Naturalis Biodiversity Center, Leiden, The Netherlands; 4 Department of Viroscience, Erasmus Medical Center, Rotterdam, The Netherlands; 5 Department of Environmental Science, Radboud Institute for Biological and Environmental Sciences, Radboud University, Nijmegen, The Netherlands

**Keywords:** Birds, *Culex pipiens*, Forest, Grassland, Habitat fragmentation, Microclimate, Usutu virus, West Nile virus

## Abstract

**Background:**

Mosquito-borne diseases are on the rise. While climatic factors have been linked to disease occurrences, they do not explain the non-random spatial distribution in disease outbreaks. Landscape-related factors, such as vegetation structure, likely play a crucial but hitherto unquantified role.

**Methods:**

We explored how three critically important factors that are associated with mosquito-borne disease outbreaks: microclimate, mosquito abundance and bird communities, vary at the landscape scale. We compared the co-occurrence of these three factors in two contrasting habitat types (forest versus grassland) across five rural locations in the central part of the Netherlands between June and September 2021.

**Results:**

Our results show that forest patches provide a more sheltered microclimate, and a higher overall abundance of birds. When accounting for differences in landscape characteristics, we also observed that the number of mosquitoes was higher in isolated forest patches.

**Conclusions:**

Our findings indicate that, at the landscape scale, variation in tree cover coincides with suitable microclimate and high *Culex pipiens* and bird abundance. Overall, these factors can help understand the non-random spatial distribution of mosquito-borne disease outbreaks.

**Graphical Abstract:**

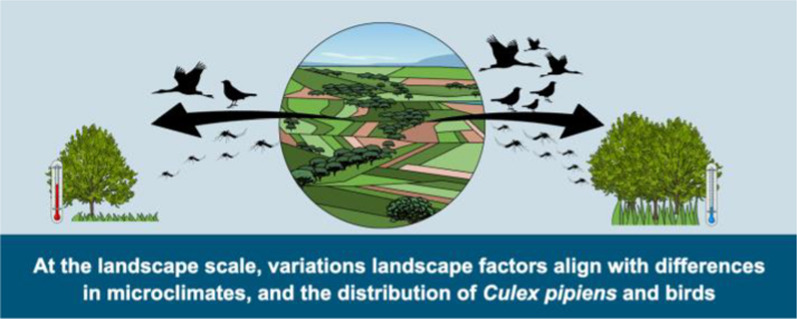

**Supplementary Information:**

The online version contains supplementary material available at 10.1186/s13071-024-06239-z.

## Background

Over the past decades, mosquito-borne diseases caused by pathogens such as viruses and parasites have become more prevalent and are spreading to new geographic areas [1, 2]. Climate change is commonly regarded as an important driving factor [3, 4]. Higher temperatures and more erratic rainfall patterns are generally linked to more beneficial conditions for mosquito development and virus replication [5, 6]. While large-scale climatic factors are strongly linked to seasonality and inter-year variation in mosquito-borne disease outbreaks, these predictors are not sufficient to explain the far from random spatial occurrence of mosquito-borne disease outbreaks. These are often confined to certain areas or emerge at the same locality year after year, suggesting that landscape factors play an important role [4, 7, 8]. This calls for a better understanding of the spatial variables that drive outbreaks.

A number of factors related to mosquito-borne disease transmission have been proposed to explain the spatially non-random occurrence of mosquito-borne disease outbreaks, such as suitable microclimate, locally present high number of disease vectors, and a high numbers of amplifying hosts [9–17]. First, *suitable microclimate* for vectors, hosts and virus replication: it is known that temperature and relative humidity play an important role in mosquito survival and virus replication [5, 6, 18–20]. At the landscape scale, high vegetation structure such as forests may play an important role in modifying microclimatic conditions such as temperature, humidity and wind speed by acting as a buffer [21, 22]. Vegetation also provides shelter against predators and stimulates non-flight movement which may lead to increased biting rates [23]. Second, *locally present high numbers of disease vectors* have been linked to spatial attributes and have been hypothesised to facilitate disease spread via the increased likelihood that an infected host gets bitten [16, 24, 25]. For example, high abundance of adult mosquitoes has previously been linked to forest fragmentation or availability of larval breeding habitat (e.g., grassy temporal water bodies) [26–28]. Third, *high numbers of amplifying hosts* for mosquito borne pathogens are important for mosquito-borne diseases transmitted by ornithophilic mosquito species, such as *Culex pipiens* and, as such, bird abundances and community composition may be of importance to its distribution and relation with pathogens [29–33]. Bird abundances can vary greatly across the landscape because of differences in habitat preferences, food availability and migratory behaviour [34–37]. Resident (sedentary) birds remain in the same area year round and can serve as both amplifying hosts and reservoirs within their home range, while migratory birds can introduce new arboviruses to a geographic area [29, 38–42]. How these factors vary across the landscape, and whether they co-occur or rather exclude each other at the landscape scale, is currently not well understood.

Here we aim to explore how these three factors, microclimate, mosquito abundance and bird communities, vary and co-occur at the landscape scale. To this end, we investigated these factors in two contrasting habitat types (forests vs. grasslands) in the relatively flat but mosaic-like landscape in the central part of the Netherlands, which mostly consists of small pockets of forest interspersed with water-rich meadows [43]. In this study we focussed on *Culex pipiens/torrentium* (hereafter *Cx. pipiens*), which is the most common and abundant mosquito (Culicidae) species in Northwestern Europe and also the primary vector for the transmission of both Usutu virus (USUV) and West Nile virus (WNV) [30, 44–50]. This unique setup allows us to study how the abovementioned factors—microclimate, mosquito abundances and host communities—vary in relative isolation of variation in macroclimatic factors.

## Methods

### Study design

**Fig. 1:**
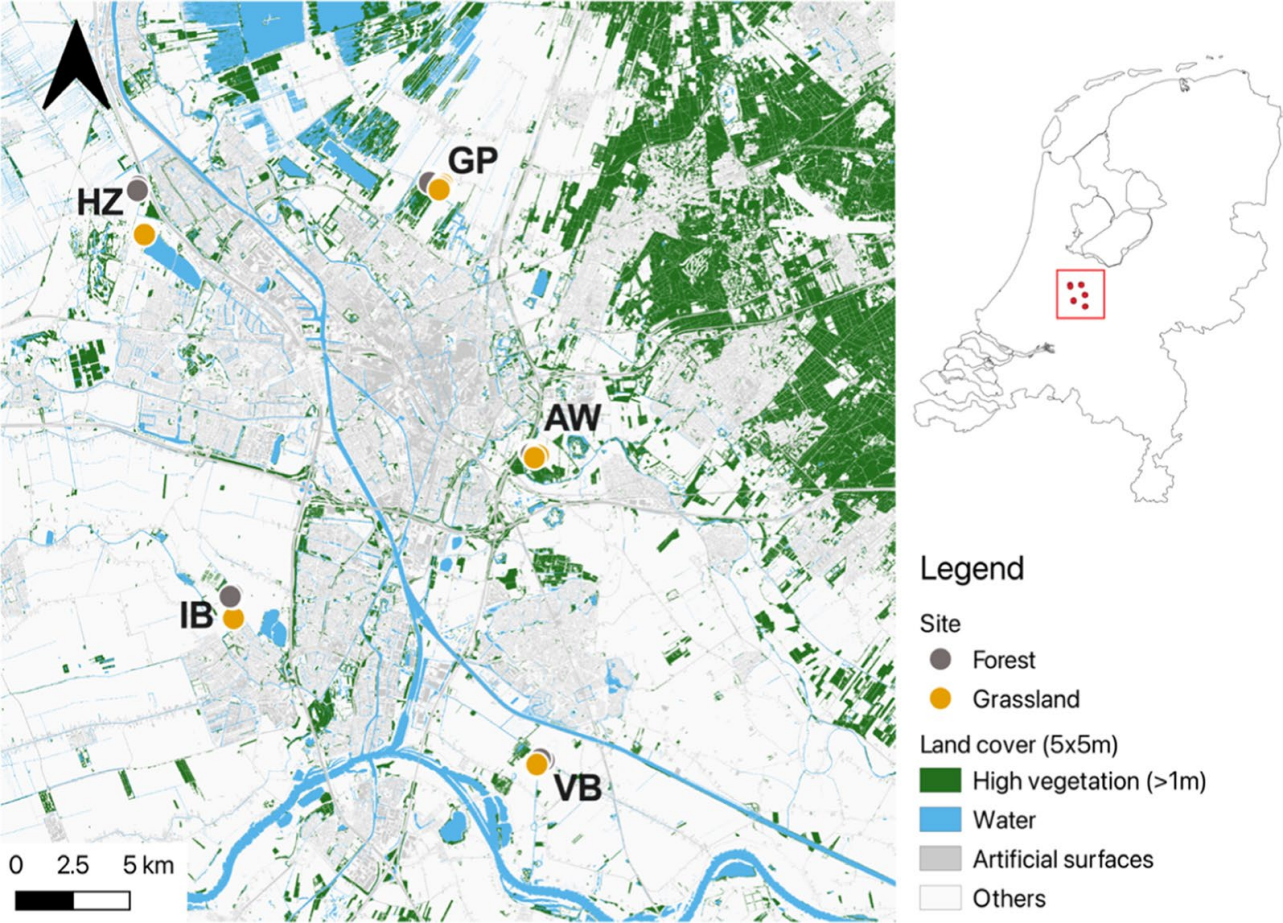
Map showing the locations of mosquito sampling in the landscape around the city of Utrecht, the Netherlands, in relation to high vegetation, water and artificial surfaces. The five locations are *Amelisweerd* (AW), *Gagelpolder* (GP), *Haarzuilens* (HZ), *IJsselsteinse Bos* (IB) and *Verdronken Bos* (VB), each with two sites, one in a forest and one in grassland

A field study was conducted between 28 June and 9 September 2021, in the topographically and climatologically homogeneous (i.e., flat) landscape of the central part of the Netherlands surrounding the city of Utrecht (Fig. 1); the altitudinal range among the sites visited was −4 m to 2.9 m relative to sea level [extracted from the EU-DEM (raster)—version 1.1, Apr. 2016; 25 m × 25 m elevation map]. The study focussed on five rural locations. Each location consisted of a paired grassland site and a forest site which were at least 100 m apart (Fig. 1) [43]. To understand how the locations vary at the landscape scale in terms of vegetation structure and landcover, we calculated a number of landscape metrics. At six sites, three in grasslands and three in forests, different aspects of the local microclimate were measured using weather stations. At all sites, the adult mosquito communities were sampled using three BG-Pro traps and the local bird communities were recorded through point-transect counts. More details regarding the study locations and sampling are provided in the following sections. All data analysis was conducted with RStudio (R version 4.1.0; R Core Team, 2021) [51]. All relevant assumptions were checked before the statistical tests were carried out. All maps were created using QGIS (version 3.16, Hannover; Development Team, 2022).

**Fig. 2:**
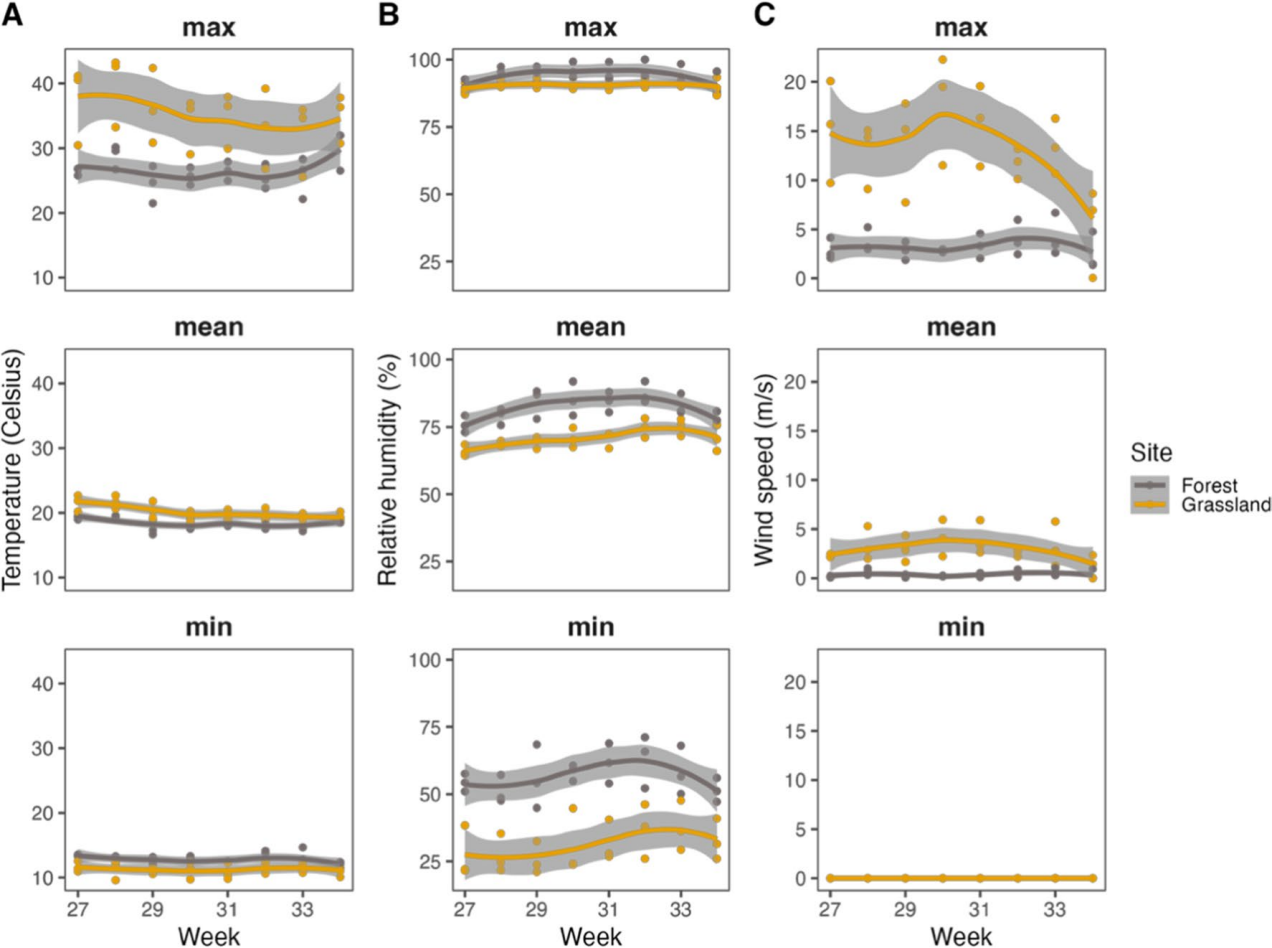
Microclimatic conditions (max, mean, min). Temperature **A**, relative humidity **B**, and wind speed **C** at three forest patches and three grasslands sites are summarised per week and weather station

### Study locations

*Amelisweerd* (AW), a former estate on the east side of Utrecht, with its forest site being an old deciduous forest with dense herbaceous vegetation and its grassland site being a large open space within the forest with agricultural grassland and abandoned bunkers. *Gagelpolder* (GP), a nature reserve on the north side of Utrecht, with its forest site being a mix of waterlogged woodland with ditches and reeds, and its grassland site being open agricultural wetland with ditches and reed vegetation. *Haarzuilens* (HZ) is a mixed nature and agricultural area with multiple forest patches connected with hedgerows in a matrix of agricultural grassland located on the west side of Utrecht. The forest site is a dense patch of vegetation, predominantly willows and other deciduous trees, while the grassland site is agricultural grassland lined with hedgerows. In 2020, WNV was detected in *Cx. pipiens* mosquitoes and in wild birds at the forest site [52]. Additionally, human cases of WNV were reported in the adjacent municipality [53]. *IJsselsteinse Bos* (IB) is a newly formed nature area for recreational purposes located on the south-west side of Utrecht, consisting of mixed deciduous forest and a dense shrub layer as forest site, with the grassland site being the surrounding agricultural grassland. *Verdronken Bos* (VB), located on the south-east side of Utrecht, is a forest in transition to a waterlogged woodland for water management purposes. Here the forest site consists of a mixed deciduous forest with a dense shrub layer and the grassland site is a meadow field with a dense herbaceous layer between two patches of forests. In the supplementary materials, photos for each forest and grassland site can be found (Additional file 1: Fig. S1).

### Calculating landscape metrics of vegetation and land cover classes

To understand how the locations vary at the landscape scale in terms of vegetation and land cover, we calculated a number of landscape metrics: (1) *Shannon diversity index*, (2) *Shannon* *evenness index*, (3) *landscape suitability score*, (4) *high vegetation cover*, (5) *high vegetation patch area* and (6) *high vegetation patch connectivity*. Landscape metrics were calculated for seven circular buffers (25, 50, 250, 500, 1000, 1500 and 3000 m) from the centroid (the middle point of the three mosquito traps) of each forest site and grassland site of the five locations, using the *landscapemetrics* R-package, based upon the 5 × 5 m LGN2021 land cover map (Additional file 1: Fig. S2) (CC BY-SA 4.0 Wageningen Environmental Research) [54]. First, we calculated the *Shannon diversity index* (shdi) and *Shannon evenness index* (shei), using all 51 land cover classes in LGN2021, to get metrics on the diversity and evenness of land cover. We then reclassified the 51 land cover classes in LGN2021 into seven new classes based on adult mosquito habitat suitability (Additional file 1: Table S1) to reduce the dimensionality and calculated a *landscape suitability score* (persl) (Additional file 1: Fig. S3). Mosquito habitat suitability score was determined based on (adult) mosquito ecological requirements, encompassing protective vegetation, presumed bird host availability, and potential availability of mosquito breeding habitats. The scoring system follows an ordinal scale and is as follows: *score 0* (others, e.g. bare ground) being not suitable for mosquitoes; *score 1* (fresh water) potential mosquito breeding habitat with predation but unsuitable for adult mosquitoes; *score 2* (grass) potential mosquito breeding habitat without predation and limited suitability for adults; *score 3* (agricultural areas) potential mosquito breeding habitat without predation, moderate availability of hosts and suitable resting habitats for adult mosquitoes; *score 4* (artificial) abundant availability of suitable breeding habitats and host but limited suitability for adults; *score 5* (high vegetation dry) low availability of breeding habitats but high availability of hosts and suitable resting habitats for adults; and *score 6* (high vegetation wet) high availability of breeding habitats, hosts and suitable resting habitats for adults (for more details on the classification see Additional file 1: Table S1). Finally, we reduced the dimensionality even further and reclassified the seven habitat suitability classes into high vegetation (1) and others (0) (Additional file 1: Fig. S4). From this we calculated the *high vegetation cover* (percentage of high vegetation; pland high), *high vegetation patch area* (mean of patch area; area mn high) and *high vegetation patch connectivity* (mean of contiguity index, more connections between patches result in larger contiguity index values; contig mn high). To test if landscape metrics differ between forest and grassland sites and between locations for all buffer distances, we performed an analysis of variance (ANOVA) followed by a multiple comparison Tukey post hoc test.

### Assessing microclimate

The microclimate at three forest and three grassland sites was monitored using a single open-source weather station for each site [55]. The weather stations were deployed on 6 July 2021 and retrieved on 9 September 2021 and recorded the following variables at five minute intervals: air temperature (°C; accuracy ± 1 °C), relative humidity (%; accuracy ± 3%) and wind speed (m/s; accuracy ± 0.1 m/s). These climatic variables are known to be of key importance for mosquito survival, development and behaviour [6, 21, 23, 56–59]. To test for differences in microclimatic conditions between forests and grasslands, we first averaged the minimum, mean and maximum values for temperature, relative humidity and wind speed on a weekly basis. Minimum wind speed was always zero and was excluded from further analysis. A linear mixed-effect model (LMEM) was run to test for differences between forests and grasslands over time for each of the three variables: temperature, humidity and wind speed and the three levels (minimum, mean and maximum). A Bartlett test was conducted to assess the homogeneity of variances between forest patches and grasslands for each of the three microclimatic variables.

### Sampling the abundances of adult *Culex pipiens* mosquitoes

Mosquitoes were collected weekly for a duration of two trapping nights at all ten sites using three carbon dioxide-baited BG Pro traps per site (Biogents GmbH, Regensburg, Germany) to capture the environmental heterogeneity at these sites [60]. To prevent any overlapping competition between traps caused by intersecting CO_2_ plumes, we spaced the traps approximately 40 m apart [61]. This was done either in a triangular pattern or a straight line, depending on the specific field conditions. To target the ornithophilic *Culex pipiens* mosquitoes, we attached the traps to a tree or pole at a height of ~1.5 m surrounded by high vegetation to provide shelter from the wind for the carbon dioxide plume [62, 63]. Carbon dioxide was generated using a modified sugar fermentation protocol, by mixing 200 g of granulated sugar, 5 g of dried *Saccharomyces cerevisiae* yeast (Fermentis, SafSpirit FD-3), 5 g of store-bought tomato paste, and approximately 1.5 L of tap water in 4 L jerrycan bags (Packforce, Jerrycan pouch) [63, 64]. The jerrycan bags were placed in the BG Pro bags and connected to the mosquito traps through silicone tubes (Ø7 mm; RubberBV, Hilversum, the Netherlands). The collected mosquitoes were stored at −20 °C until separated by sex and the female mosquitoes were identified morphologically based on the characteristics outlined in Becker et al. [56]. *Culex pipiens* s.l. and its sibling species *Cx. torrentium* are challenging to differentiate reliably based on morphological characteristics of adult females and were therefore grouped together and referred to as *Cx. pipiens* [56, 65, 66].

To test for the overall differences in abundances of *Cx. pipiens* between forests and grasslands, we performed a generalised linear mixed model (GLMM) with a negative binominal distribution, using maximum likelihood (ML) and the BOBYQA optimiser. For the response variable, we aggregated the mosquito count data per site, location and week. For explanatory variables, we evaluated the independent and interactive effects of site (forest versus grassland) and location (to capture the effect of landscape). Week was included as a random effect (to avoid pseudo-replication).

To evaluate the role of landscape on mosquito abundances, especially that of habitat and high woody vegetation structure, we replaced location by each of the before mentioned landscape metrics (*Shannon diversity index*, *Shannon evenness index*, *landscape suitability score*, *high vegetation cover* and *high vegetation patch area and high vegetation patch connectivity*) for each of the seven buffer distances (25, 50, 250, 500, 1000, 1500 and 3000 m) in the GLMM. We did not rescale the landscape metrics. To test the effect of the individual explanatory variables (i.e., site, location, metric) and their interactions we employed a Wald post hoc test. To assess the different models in terms of explained variance we used the Akaike Information criterion (AIC) and Bayesian information criterion (BIC) to rank each model.

### Assessing bird abundances and community composition

Birds within hearing distance of each of the three mosquito traps per site were counted using a modified point transect count protocol [67]. Birds were counted during a 5 min period at each of the mosquito trap sites as points, and along a standardised route between the traps as transects. Counts were performed five times starting from 05:20 am (Central European Time) at each of the sites, with the order of the sites being randomised over two consecutive weeks. Data were collected by observing the birds’ vocal sounds and, when possible, through visual observation. To test for differences in bird abundances between forests and grasslands, a paired *t*-test by location was conducted. To test for differences in bird community between forests and grasslands, a presence/absence Bray–Curtis distance matrix between the different data points was calculated and a non-metric multidimensional scaling (NMDS) analysis was run on the distance matrix. A permutational multivariate analysis of variance (PERMANOVA) was performed with the Bray–Curtis distances between sites as the response variable and forest versus grassland as explanatory variable. Inclusion of location in the PERMANOVA did not explain much variation and was omitted. Each bird species was then categorised based on its migration behaviour [34]. To test if bird communities differ between grasslands and forests in terms of migratory behaviour (sedentary birds, short-distance and long-distance migratory birds), a Chi-squared test was performed followed by a post hoc test with Bonferroni correction for multiple comparisons.

### Assessing co-occurrence among landscape metrics, microclimate variables, *Culex pipiens* and bird communities

We employed Spearman rank correlation tests assess the co-occurrence among landscape metrics, microclimate variables, *Culex pipiens* abundances and bird communities. Due to the non-synchronous nature of our data collection for microclimate, mosquitoes and birds, we omitted the temporal dimension. We summarised the minimum, mean and maximum values of the microclimate variables (temperature, humidity and wind speed) per site (forest versus grassland), since we did not have weather stations at all locations. *Culex pipiens* abundances and the bird community (including total bird abundance, the abundance of sedentary, short- and long-distance migratory birds) were summarised per site and location. The landscape metrics (*Shannon diversity index*, *Shannon evenness index*, *landscape suitability score*, *high vegetation cover*, *high vegetation patch area* and *high vegetation patch connectivity*) for each of the seven buffer distances (25, 50, 250, 500, 1000, 1500, and 3000 m) were already formatted per site and location.

## Results

### Differences in landscape metrics of vegetation and land cover classes

The *Shannon diversity index* and *Shannon evenness index*, which encompass all LGN2021 land cover classes, are statistically significant between different locations starting at buffer distances of 1000 m but not between forest and grassland sites (Additional file 1: Fig. S5; Table. S2–S3). Conversely, forest and grassland sites are statistically significant different for *landscape suitability score* (Additional file 1: Fig. S5; Table S4) and *high vegetation cover* (Additional file 1: Fig. S6; Table S5) at the small scale for buffer distances ranging from 25 to 50 m and between locations at the large scale starting a buffer distances of 1000 m. *High vegetation patch area* is statistically significant different between forest and grassland sites at 250 m and between locations starting at a buffer distance of 500 m (Additional file 1: Fig. S6; Table S6). *High vegetation patch connectivity* is statistically significant different between locations starting at a buffer distance of 1000 m and does differ between forest and grassland sites (Additional file 1: Fig. S6; Table S7). Interestingly, we observe that for most landscape metrics the locations *IJsselsteinse Bos* (IB) and *Verdronken Bos* (VB) are statistically indistinguishable.

### Differences in micro-climatic conditions between forest patches and grasslands

The microclimate in forest patches and grasslands showed significant differences in temperature (mean, maximum), relative humidity (minimum, mean) and wind speed (mean, maximum). Maximum temperature in forest patches was on average 8.6 °C lower (26.7 versus 35.3 °C) (LMER, *t*-value = −2.81, *df * = 4, *Ρ* < 0.05; Fig. 2A) and mean temperature was 1.7 °C lower (18.5 versus 20.2 °C) (LMER, *t*-value = −3.61, *df* = 4, *Ρ* < 0.05; Fig. 2A) compared with grasslands, but minimum temperatures were on average 1.5 °C higher in forest patches (12.8 versus 11.3 °C) (LMER, *t*-value = 3.23, *df * = 4, *Ρ* < 0.05; Fig. 2A). For humidity we found the opposite pattern: forest patches maintained 25.4% higher levels of minimum relative humidity (56.7% versus 31.3%) (LMER, *t*-value = 4.31, *df* = 4, *Ρ* < 0.05; Fig. 2B) and 11.5% higher levels of mean relative humidity (82.2% versus 70.7%) (LMER, *t*-value = 4.66, *df* = 4, *Ρ* < 0.01; Fig. 2B); maximum relative humidity was 3.5% higher in forest patches (94.0% versus 90.5%) but not statistically significant (LMER, *t*-value = 3.50, *df* = 4, *Ρ* = 0.175; Fig. 2B). The pattern for wind speed was very similar as for temperature. Grasslands were exposed to stronger wind gusts as indicated by the higher maximum wind speed (3.3 m/s versus 13.2 m/s) measured in these areas (LMER, *t*-value = −9.31, *df* = 4, *Ρ* < 0.01; Fig. 2C). Forest patches had a lower mean wind speed compared to grasslands (0.4 m/s versus 3.0 m/s) (LMER, *t*-value = − 8.08, *df* = 4, *Ρ* < 0.01; Fig. 2C). When examining the variance in microclimatic conditions between forests and grasslands, we observed that grasslands have greater variability in temperature (Bartlett test, *K*^2^ = 20.83, *df* = 1, *Ρ* < 0.001), humidity (Bartlett test, *K*^2^ = 12.263, *df* = 1, *Ρ* < 0.001) and wind speed (Bartlett test, *K*^2^ = 100.69, *df* = 1, *Ρ* < 0.001) compared with forest patches.

**Fig. 3:**
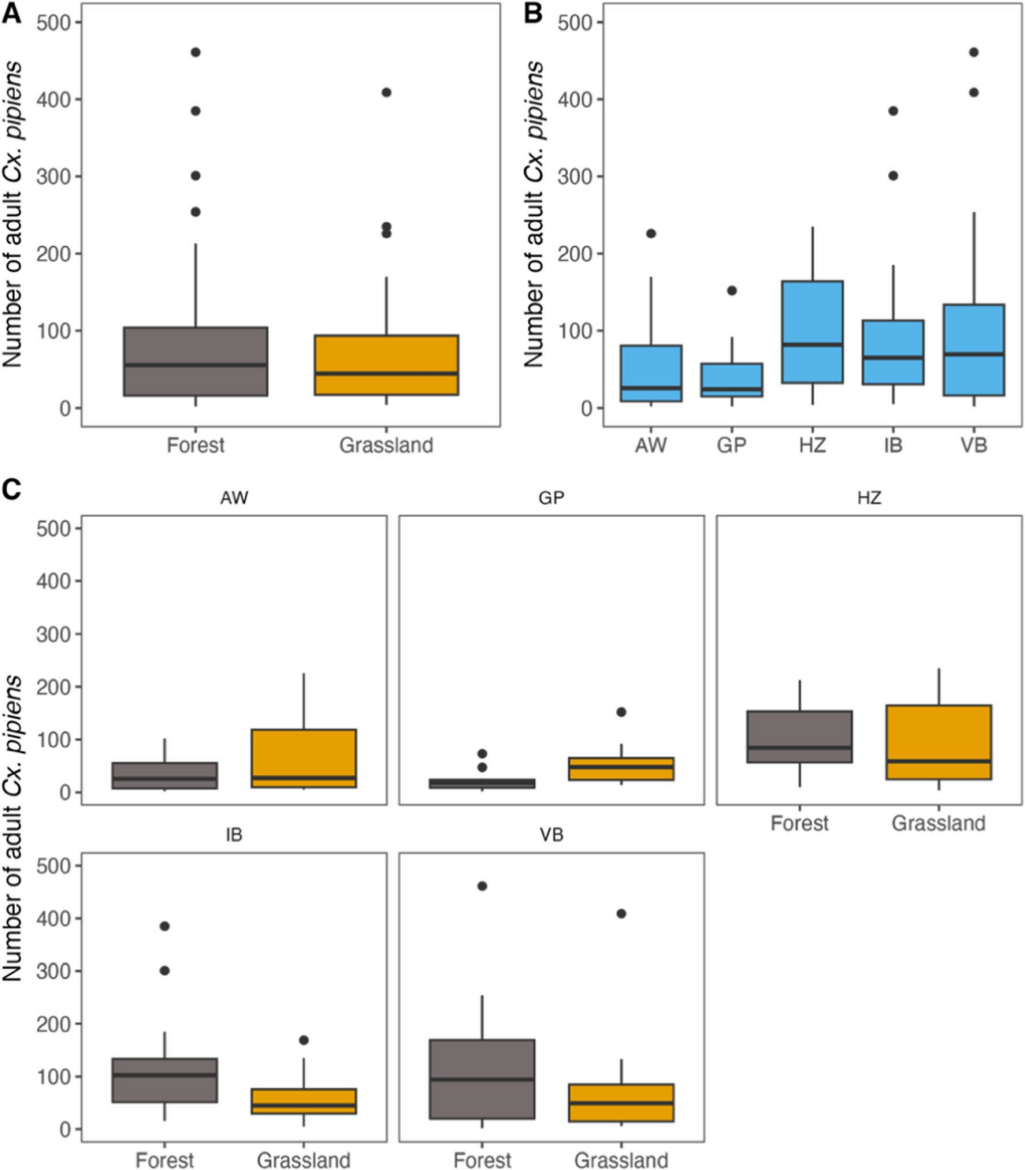
The total number of captured female *Culex pipiens* mosquitoes per week at each of the five forest patch sites and five grassland sites **A** per location **B** and per paired sites **C**. See Table S9–16 for results of the statical analysis

### Mosquito abundances between forest patches and grassland

We captured a total of 9493 female mosquitoes and identified ten species, with *Cx. pipiens* being the dominant species (~96%) (Additional file 1: Table S8). We trapped on average 15% more adult mosquitoes in forest sites compared to grassland sites. We first fitted a null model with a constant (intercept-only) term to predict the number of adult mosquitoes, the model incorporated week as a random effect, and the intercept was estimated at 4.03 [95% confidence interval (CI) [3.57, 4.49], *p* < 0.001]. We then fitted a GLMM to test if the difference in the number of adult mosquitoes was statistically significant between forest patches and grasslands. The model had a substantial explanatory power with an *R*^2^ of 0.73, of which 0.22 is related to the fixed effects (e.g. forest versus grasslands, location and their interaction).When we looked at the effect of site (forest versus grasslands) on number of adult mosquitoes, we observed that this difference was statistically significant (Wald test, *χ*^2^ = 4.441, *df* = 1, *Ρ* = 0.035; Fig. 3A). Also, when we looked at the effect of location on number of adult mosquitoes, we observed that this difference was statistically significant (Wald test, *χ*^2^ = 71.595, *df* = 1, *Ρ* < 0.001; Fig. 3B), as well as the interaction of location with site (Wald test, *χ*^2^ = 32.770, *df* = 1, *Ρ* < 0.001; Fig. 3B). However, when we performed a pairwise comparison between site (forest versus grasslands) we observed that the difference was statistically non-significant [Tukey post hoc test, estimate −0.036, standard error (SE) 0.119, *df* = 1, *z*-ratio  −0.299, *Ρ* = 0.765; Fig. 3A]. Also, when looking at the differences between site (forest versus grasslands) per location, we observed that with the exception of the *Gagelpolder* (GP), all locations forest and grassland sites were statistically indistinguishable (Fig. 3C; Additional file 1: Table S9). When we performed a pairwise comparison between locations, we observed that the locations, in terms of number of adult mosquitoes, can be divided into two groups, the low abundance group (AW, GP) and the high abundance group (HZ, IB, VB) (Fig. 3B; Additional file 1: Table S10).

### Differences in mosquito abundances between grassland and forest patches in relation to landscape metrics

Although some forest sites exhibited markedly higher mosquito numbers than grassland sites, and vice versa, none of these differences could be explained by the contrast between forest and grassland alone, but inclusion of landscape metrics resulted in a major improvement of the model performance. The model with the *landscape suitability score* metric and a buffer distance of 500 m explained the most variance, followed by models with the *high vegetation cover* metric and with buffer distances of 500, 1000 and 1500 m and the model with the *Shannon evenness index* metric and a buffer distance of 3000 m. For all these models the metric has a negative relation with the abundance of mosquitoes. Interestingly we observe that models containing the *landscape suitability score* metric with a buffer distance larger than 50 m were clustered around the basic model in terms of explained variance expressed in AIC and BIC (Additional file 1: Fig. S7). Also, these models have a negative relation with the abundance of mosquitoes. Models with the other metrics and buffer distances did not perform better than the basic model in terms of explaining the variance in mosquito abundances. Furthermore, multiple post hoc Wald chi-squared tests of our models indicated that the abundance of mosquitoes is statistically significantly related with all landscape metrics at most buffer distances, with the exception of *high vegetation patch area* and *high vegetation patch connectivity*, and that the relation with the abundance of mosquitoes was often negative (Additional file 1: Table S11–16).

### Differences in bird communities between forest patches and grasslands

**Fig. 4:**
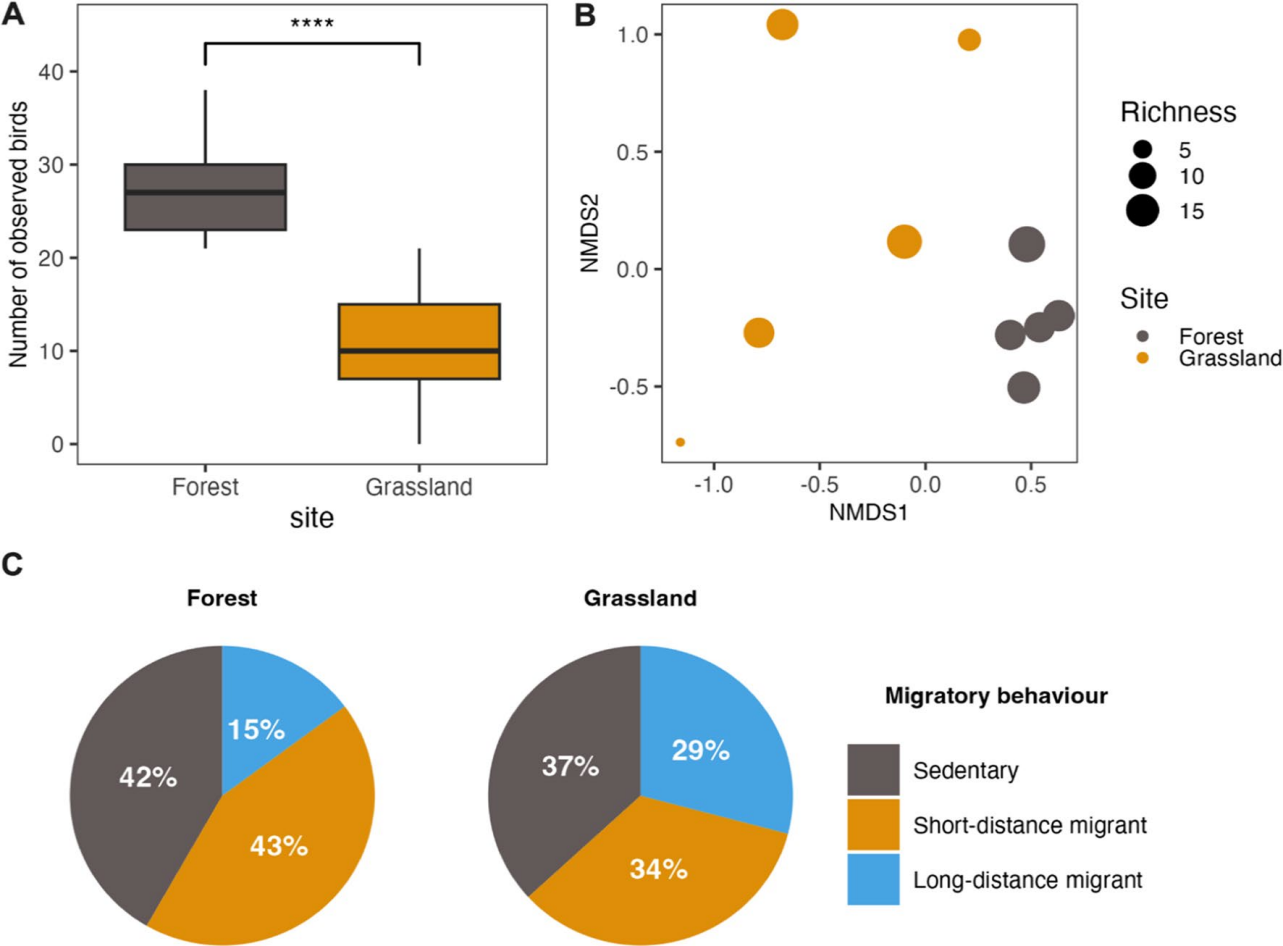
The observed bird counts at both the forest and grassland sites **A**, with differences in community composition displayed in a non-metric multidimensional scaling graph **B** based on a Bray–Curtis presence/absence distance matrix (linear fit *R*^2^ = 0.986, non-metric fit *R*^2^ = 0.997). The ratios of observed birds at grasslands and forest patches that are sedentary birds, short-distance migratory bird and long-distance migratory birds **C**

We recorded 566 birds from 45 species (Additional file 1: Table S17). In forest sites, the most commonly observed species were *common chiffchaff* (*n* = 75), *common blackbird* (*n* = 67), *Eurasian wren* (*n* = 45), *Eurasian blackcap* (*n* = 42) and *common chaffinch* (*n* = 41). In grassland sites, the most frequently observed species were *reed bunting* (*n* = 21), *sedge warbler* (*n* = 17), *Eurasian reed warbler* (*n* = 15), *common blackbird* (*n* = 13) and *barn swallow* (*n* = 11). The bird community differed in both the number of observed birds (paired *t*-test, *t*-value of − 8.136, *df* = 14, *Ρ* < 0.001; Fig. 4A) and in community composition, with a higher species richness and greater diversity in forests compared to grasslands (adosin, *R*^2^ = 0.41, *df* = 9, *F* = 5.533, *Ρ* < 0.05; Fig. 4B). We found that the ratio of bird migratory behaviour differed between forests and grasslands (*χ*^2^ = 23.301, *df* = 2, *Ρ* < 0.001; Fig. 4C). A post hoc test revealed that there was no statistically significant difference in ratio between forests and grasslands in terms of sedentary birds (*χ*^2^ = 23.301, *df* = 2, *Ρ* = 1.00) and short distance migratory birds (*χ*^*2*^ = 23.301, *df* = 2, *Ρ* = 0.08). However, we did observe that ratio long distance migratory birds were relatively larger in grasslands (*χ*^*2*^ = 23.301, *df* = 2, *Ρ* < 0.001).

### Co-occurrence among landscape metrics, microclimate variables, *Culex pipiens* and bird communities

**Fig. 5:**
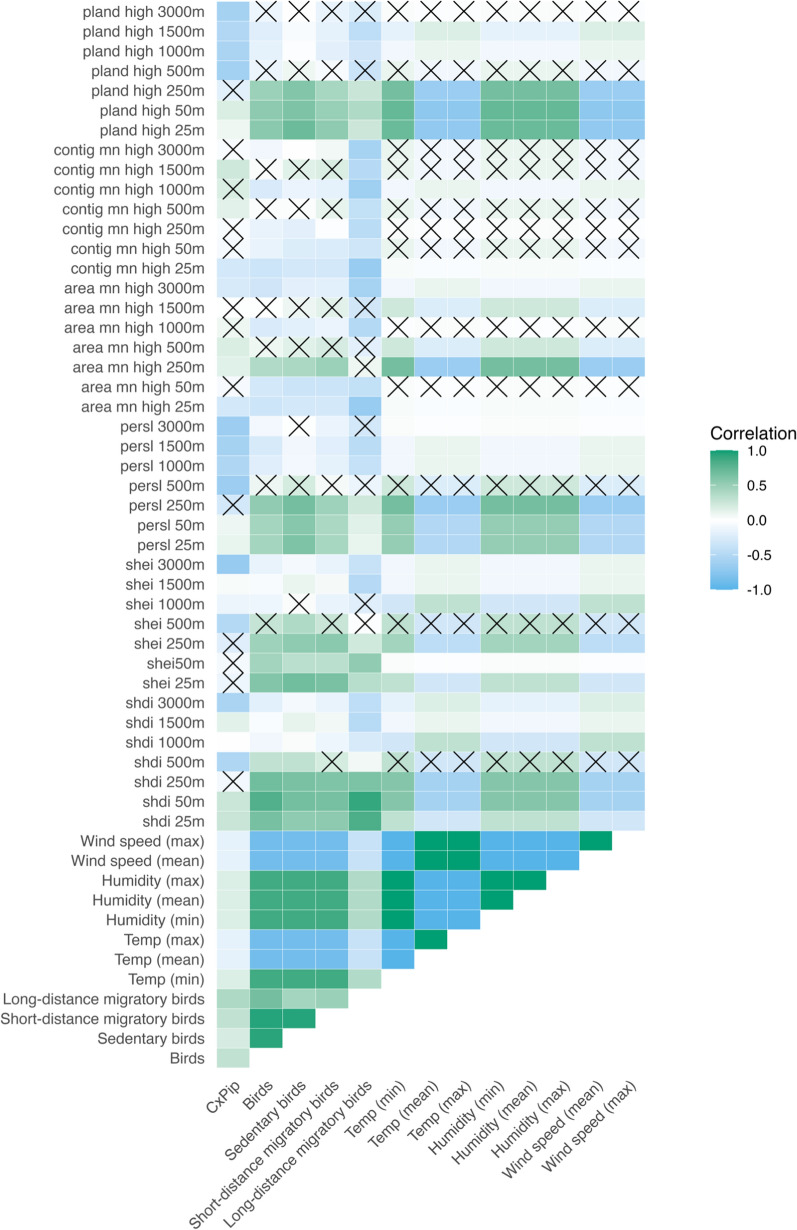
Spearman rank correlation between landscape metrics, microclimate variables, *Culex pipiens* and bird communities. Cells with an ‘X’ indicate a statistically non-significant relationship

*Culex pipiens* and bird abundances—both total and categorised as sedentary, short- and long-distance migratory birds—are positively correlated. Furthermore, *Culex pipiens* and bird abundances are positively correlated with minimum temperature, humidity and negatively correlated with maximum and mean temperature and wind speed (Fig. 5). Among the landscape metrics, *high vegetation cover* and *landscape suitability score* showed a similar pattern in terms of *Culex pipiens* and bird abundances, having a positive correlation at small buffer distances and a negative beyond 250 m. However, the correlation between *Culex pipiens* abundances with microclimate variables and landscape metrics are notably weaker compared to bird abundances.

## Discussion

The central aim of this paper was to investigate how microclimate, mosquito abundance and bird communities vary and co-occur at the landscape scale, which all play a role in the transmission cycle of mosquito-borne pathogens for which birds serve as reservoir hosts. In this study, we used a design with two contrasting dominant habitat types in the rural landscape of the central part of the Netherlands. Our results show that forest patches provide a more sheltered microclimate and a higher overall abundance of birds. When accounting for differences in landscape characteristics, we also observed that the number of mosquitoes was higher in isolated forest patches. Overall, these results suggest that forest patches may harbour more favourable conditions for adult mosquitoes and birds. Each of the focal factors of this study—microclimate, mosquitoes and birds—shows a unique response to the heterogeneous landscape and they may in the end not all be equally important. This is discussed further below.

### Forest patches provide favourable microclimate conditions for mosquitoes and birds

Our results confirm that in high woody vegetation, such as the forest patches, microclimates are overall cooler, less windy, and more humid than the surrounding low vegetation grassland areas [22]. Temperature plays a significant role in the physiology, development and behaviour of both mosquitoes and birds [58, 68–70]. Likewise, high humidity and lower wind speeds, as observed in the forest patches, has previously been found to increase mosquito survival and reproduction [5, 6, 18]. Adult mosquitoes are fragile animals which dry out very quickly and are easily overwhelmed by strong gusts of wind [23, 71]. Wind may also impact host-seeking behaviour, which depends on mosquito olfactory senses over long distances, making it necessary for mosquitoes to sense these compounds downwind [57, 61]. In order for mosquitoes to navigate towards these sources, the prevailing wind speed must be lower than their average flight speed of 1 m/s [71]. Denser high vegetation areas may protect mosquitoes from the wind and may facilitate mosquito host-seeking behaviour by limiting bird flight and encouraging non-flight locomotion of mosquitoes via branches [23]. Forest patches may also provide a refuge for adult mosquitoes and birds during heatwaves and droughts [14, 72]. Depending on the virus-specific optimal temperatures for replication, we speculate that the microclimate in these forest patches likely provides more beneficial conditions for virus transmission [5, 18, 73]. Conversely, low vegetation, such as (irrigated) grasslands, lacks the ability to modulate these climatic factors. However, it can provide a suitable habitat for mosquito breeding, as it can support isolated grassy temporal water bodies, with food (via soil nutrients and microbial community) and vegetation (grass) that provides protection from predators [22, 26]. Our results show that small forest patches in a mostly open grassland could potentially aggregate large numbers of birds and mosquitoes, facilitating their interaction and possibly increasing biting rates [14, 74–76]. However, this should be investigated in more detail with blood-meal analysis.

### Landscape characteristics modify mosquito abundances in forest patches and grasslands

Previous studies have found clear associations between vegetation structure and adult mosquito abundances for a number of mosquito species, including *Cx. pipiens* [21, 77–81]. Despite the more favourable (microclimatic) conditions, we only observed on average 15% more mosquitoes in forest sites compared to grassland sites. At two locations (AW, GP), we trapped more mosquitoes at the grassland site than the corresponding forest site. Interestingly, we captured fewer mosquitoes overall at these locations than at others. Conversely, for two locations (IB, VB) we can see a clear but non-significant difference between the two contrasting habitats, with more mosquitoes observed at the forest sites compared with the grassland sites. Moreover, these locations (IB,VB) were statistically indistinguishable for most landscape metrics. This begs the question, why did we observe such a small difference between contrasting habitats despite these large differences in vegetation structure? We can think of two possible non-mutually exclusive explanations for this.

The first explanation is that we may have sampled the same mosquito community at our contrasting paired sites, and that variation in mosquito abundance among location was mostly related to landscape level characteristics and differences therein. While mosquitoes themselves are small, the scale at which they interact with the environment may not be. We assumed that the (minimum) distance of 100 m between our forest and grassland sites was large enough to avoid individual mosquitoes moving between sites. However, several studies suggest that mosquito dispersal distance may be larger, particularly for *Culex* spp., ranging from a few metres to several kilometres [21, 82]. Interestingly, our models that take landcover heterogeneity at different buffer distances into account, explain significantly more variation in mosquito abundance than site (forest versus grassland) alone. For most of these landscape metrics we found a negative relation, indicating that increasing tree cover (which was the basis for most of our landscape metrics) and decreasing landscape heterogeneity (Shannon diversity and evenness indices) have an overall negative effect on the abundances of mosquitoes. Indeed, when we look at the total mosquito abundance, we observe that at the small scale mosquito abundance is positively correlated with high vegetation cover and a negatively at a larger scale (> 250 m). A similar pattern is visible for high vegetation patch area, where we see that at a small scale (< 500 m), a larger patch area is positively correlated with mosquito abundance, and a negatively beyond 500 m.

The locations AW and GP are both characterised by a large continuous forest (> 500 m), with open grassland within for AW and surrounded by grassland for GP. At these locations we found a lower overall abundance in mosquitoes and more mosquitoes at grassland sites than forest sites. The locations IB and VB are both characterised as a small (< 500 m) isolated forest surrounded by agricultural grassland. At these locations we found a higher overall abundance of mosquitoes compared to GP and AW, and a clear but non-significant difference between the two contrasting habitats. The location HZ is characterised by multiple patches of forest (< 500 m) that are interconnected by hedgerows. This coincides nicely with our high vegetation patch connectivity results, which has a negative effect on mosquito abundance at the small scale and a positive effect beyond 500 m. Overall, we observed the most mosquitoes at HZ, with higher abundances in forest patches than in grassland, but not significantly so. These results suggest that landcover heterogeneity and confounding environmental drivers of mosquito abundance are more important than previously thought and have a distinct and opposite effects at different scales.

Our second explanation for the small observed differences between forest and grassland sites is linked to the inherent constraints regarding trap placement, which might have reduced our ability to detect differences between contrasting habitat types. For traps to attract mosquitoes, they need to be placed in a sheltered spot, such that a carbon dioxide plume can develop [61, 63]. This means that regardless of landscape type, traps need to be placed in sheltering vegetation, such as a small hedgerow, under a solitary tree with surrounding shrubs, or in a patch of reeds. In turn, such microhabitats might already provide sufficient protection against adverse climatic conditions for mosquitoes to aggregate. Hypothetically, the open areas in grassland habitats could be largely devoid of mosquitoes, but CO_2_-based trapping methods may be unable to measure this [56, 63]. Anecdotal observations in the field support this hypothesis. While in small forest patches mosquitoes were omnipresent, including on exposed parts of the body (face, arms), in larger forest patches mosquitoes were clustered in certain areas. Conversely, in grasslands, mosquitoes were only observed in small patches of vegetation where traps were placed.

While both explanations may (at least partly) explain the absence of a larger effect, our results do show notable differences in mosquito abundances between contrasting landscapes, thus highlighting the strength of the association between landscape and mosquito abundance.

### Substantial differences in bird abundances and species composition between forests and grasslands

Compared with grassland sites, forest sites exhibited a significantly higher overall bird abundance. Furthermore, forest sites had a distinctly different species composition compared with grassland sites. Species composition in all forest sites was relatively similar, while large differences in species composition were found between the grassland sites.

The most abundant species at forest sites are the *common chiffchaff*, *common blackbird*, *Eurasian wren*, *Eurasian blackcap* and *common chaffinch*. All bird species at forest sites are known residents of forests and or all susceptible to both or either WNV and/or USUV [34, 44, 83, 84]. The most abundant species at grassland sites are the *reed bunting*, *sedge warbler*, *Eurasian reed warbler*, *common blackbird* and *barn swallow*. All species are known residents of grassland and all except the *reed buntin* and *Eurasian reed warbler* are known to be susceptible to both or either WNV and/or USUV [34, 44, 83, 84]. Overall, in both forests and grasslands, the most commonly observed species belong to the Passeriformes order. While studies have shown that passerines can be highly susceptible to WNV and USUV, not all species are known to be susceptible or can have different within-species susceptibility depending on specific behaviour [29, 44, 85, 86]. For example, it has been shown that the innate immunity of the *common blackbird* is reduced when they migrate, which might relate to the hardship experienced during migration [87]. We also found that the bird communities differ significantly in terms of migratory behaviour, especially for long-distance migration. Forests harbour higher overall abundances of sedentary and migratory birds compared to grasslands, with sedentary birds utilizing these forests throughout the year, while migratory birds use these forests as resting and breeding grounds during migration [88–90]. These high bird abundances might increase the likelihood that an infected mosquito encounters an arbovirus susceptible bird. Grasslands are, relative to forests, home to more long-distance migratory birds, which might play a role in introducing arboviruses to an area from their overwintering habitats in the southern hemisphere [29, 38–41].

Interestingly we found that the *common blackbird* occurs in both forests and grasslands. This species with its large home range might play an important role in spreading arboviruses between and within forests and grasslands, which then might act as stepping stones in spreading a disease across a larger area, including to urban areas, once it has been introduced [29, 91]. Despite the fact that forests host higher abundances/densities of birds compared to grasslands, it might not mean that individual birds in these forests are more at risk of arbovirus infection, due to what is known as the ‘encounter-dilution effect’, e.g. the chance that an infected mosquito encounters an individual bird [92]. Nevertheless, our results have shown that high abundances of birds and mosquitoes do co-occur with suitable microclimate conditions in small, isolated forest patches, which might translate in higher overall infection rates in both mosquitoes and birds at a population level. However, this must be evaluated in an area with active arbovirus circulation.

## Conclusions

Together, the results of this study show that microclimates, as well as the local abundance and composition of birds, strongly co-vary at the landscape scale. Perhaps unexpectedly, we found that the contrasting habitat types alone do not explain the variation in the abundance of mosquitoes, but that landscape metrics on multiple scales related to differences in tree cover also explain part of the variation. Although microclimate and the abundance of *Cx. pipiens* and birds have all previously been individually associated with habitat types, this is the first study that studies co-occurrence of all three factors. Overall, our results suggest that at the landscape scale, variations in tree cover, especially in small forest patches, coincide with suitable microclimates and high *Cx. pipiens* and bird abundances. These factors can help understand the strongly non-random spatial distribution of mosquito-borne disease outbreaks.

### Supplementary Information

**Additional file 1: Figure S1:** Study design with five locations, each consisting of a paired grassland site and a forest patch site. The five locations are: *Amelisweerd* **A**, *Gagelpolder* **B**, *Haarzuilens* **C**, *IJsselsteinse Bos* **D** and *Verdronken Bos* **E**. **Figure S2.** LGN2021 5 m × 5 m land cover map (CC BY-SA 4.0 Wageningen Environmental Research). **Figure S3.** Reclassification of LGN2021 map into seven new classed based upon adult mosquito habitat suitability (for more details on the classification see Table S1). **Figure S4.** Reclassification of landscape suitability map into high vegetation and others. **Figure S5.** Landscape metrics for each of the five locations and per forest patch and grassland site within a 25, 50, 250, 500, 1000, 1500 and 3000 metres buffer from the centroid of the site. **Figure S6.** Landscape metrics for each of the five locations and per forest patch and grassland site within a 25, 50, 250, 500, 1000, 1500 and 3000 m buffer from the centroid of the site. **Figure S7.** Model performance to explain spatial pattern in mosquito abundance based on Akaike information criterion (AIC) and Bayesian information criterion (BIC) values. **Table S1.** LGN2021 5 m × 5 m land cover map (CC BY-SA 4.0 Wageningen Environmental Research). **Table S2.** Summary of ANOVA tests results. **Table S3.** Summary of ANOVA tests results. **Table S4.** Summary of ANOVA tests results. **Table S5.** Summary of ANOVA tests results. **Table S6.** Summary of ANOVA tests results. **Table S7.** Summary of ANOVA tests results. **Table S8.** Sampled mosquito populations at forest and grasslands sites. **Table S9.** Summary of a pairwise multiple comparison between Tukey post hoc test results in which the differences in the number of mosquitoes between site (forest versus grassland) is evaluated per location. **Table S10.** Summary of a pairwise multiple comparison between Tukey post hoc test results in which the differences in the number of mosquitoes is evaluated among locations. **Table S11.** Summary of type III Wald chi-squared tests results for various generalized linear mixed models. **Table S12.** Summary of type III Wald chi-squared tests results for various generalized linear mixed models. **Table S13.** Summary of type III Wald chi-squared tests results for various generalized linear mixed models. **Table S14.** Summary of type III Wald chi-squared tests results for various generalized linear mixed models. **Table S15.** Summary of type III Wald chi-squared tests results for various generalized linear mixed models. **Table S16.** Summary of type III Wald chi-squared tests results for various generalized linear mixed models. **Table S17.** Sampled bird population at forest and grasslands sites. In total, 566 birds were observed belonging to 45 different species.

**Additional file 2: **Data landscape factors, containing mosquito trapping, bird observation and microclimate data.

## Data Availability

All data are available in Additional File 2.
