# Supplementary material for: Landscape level associations between birds, mosquitoes and microclimates: possible consequences for disease transmission?
Source: Parasit Vectors. 2024 Mar 26;17:156. doi: 10.1186/s13071-024-06239-z (PMC10964671; doi:10.1186/s13071-024-06239-z)
Supplement: Supplementary file 1 — Additional file 1: Figure S1: Study design with five locations, each consisting of a paired grassland site and a forest patch site. The five locations are: Amelisweerd A, Gagelpolder B, Haarzuilens C, IJsselsteinse Bos D and Verdronken Bos E. Figure S2. LGN2021 5 m × 5 m land cover map (CC BY-SA 4.0 Wageningen Environmental Research). Figure S3. Reclassification of LGN2021 map into seven new classed based upon adult mosquito habitat suitability (for more details on the classification see Table S1). Figure S4. Reclassification of landscape suitability map into high vegetation and others. Figure S5. Landscape metrics for each of the five locations and per forest patch and grassland site within a 25, 50, 250, 500, 1000, 1500 and 3000 metres buffer from the centroid of the site. Figure S6. Landscape metrics for each of the five locations and per forest patch and grassland site within a 25, 50, 250, 500, 1000, 1500 and 3000 m buffer from the centroid of the site. Figure S7. Model performance to explain spatial pattern in mosquito abundance based on Akaike information criterion (AIC) and Bayesian information criterion (BIC) values. Table S1. LGN2021 5 m × 5 m land cover map (CC BY-SA 4.0 Wageningen Environmental Research). Table S2. Summary of ANOVA tests results. Table S3. Summary of ANOVA tests results. Table S4. Summary of ANOVA tests results. Table S5. Summary of ANOVA tests results. Table S6. Summary of ANOVA tests results. Table S7. Summary of ANOVA tests results. Table S8. Sampled mosquito populations at forest and grasslands sites. Table S9. Summary of a pairwise multiple comparison between Tukey post hoc test results in which the differences in the number of mosquitoes between site (forest versus grassland) is evaluated per location. Table S10. Summary of a pairwise multiple comparison between Tukey post hoc test results in which the differences in the number of mosquitoes is evaluated among locations. Table S11. Summary of type III Wald chi-squared tests results [file 13071_2024_6239_MOESM1_ESM.docx]

**Supplementary information**

Landscape level associations between birds, mosquitoes and microclimates: possible consequences for disease transmission?**
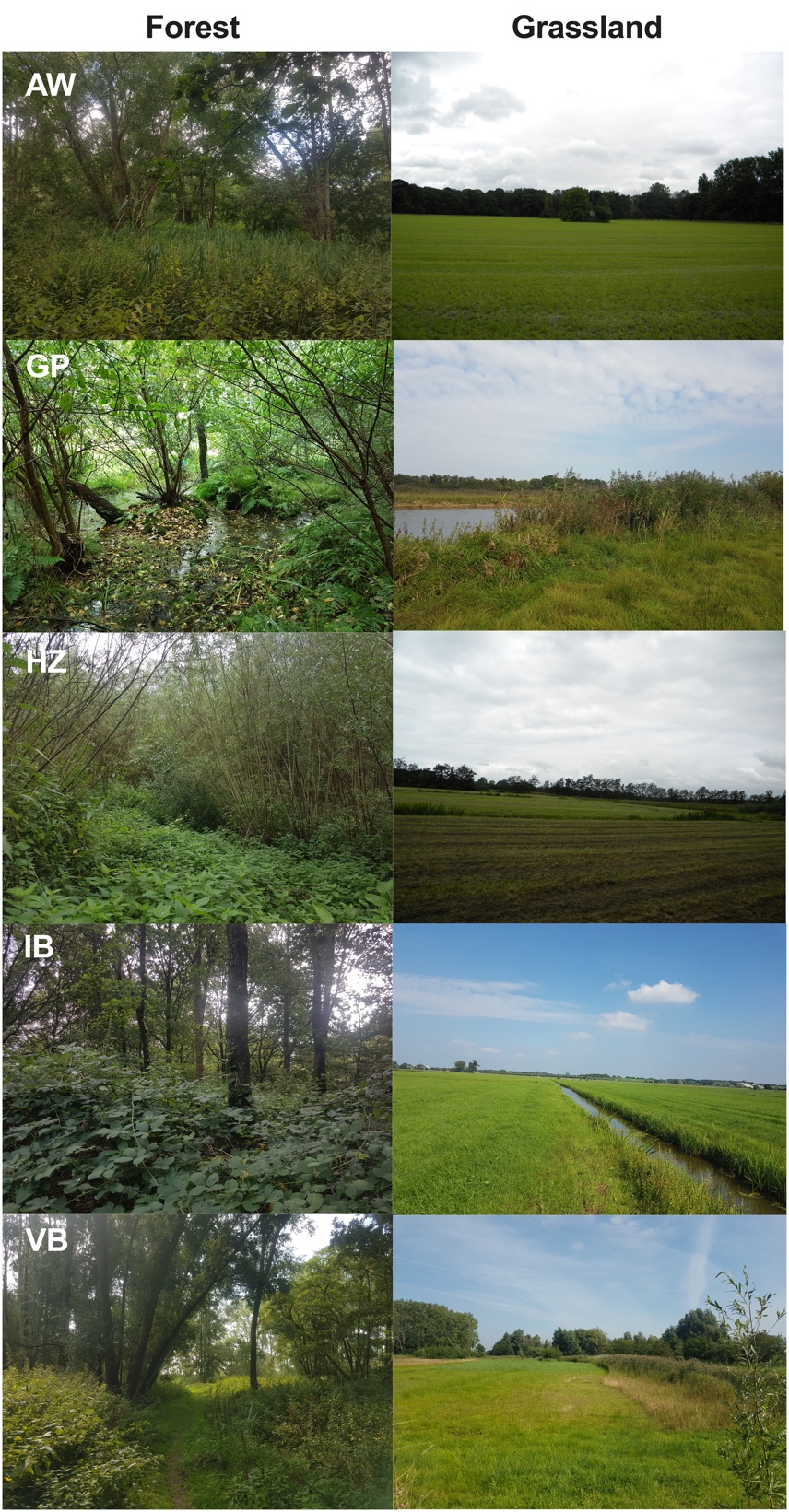
**

**Figure S1:** Study design with five locations, each consisting of a paired grassland site and a forest patch site. The five locations are: *Amelisweerd* (A), *Gagelpolder* (B), *Haarzuilens* (C), *IJsselsteinse Bos* (D), and *Verdronken Bos* (E).


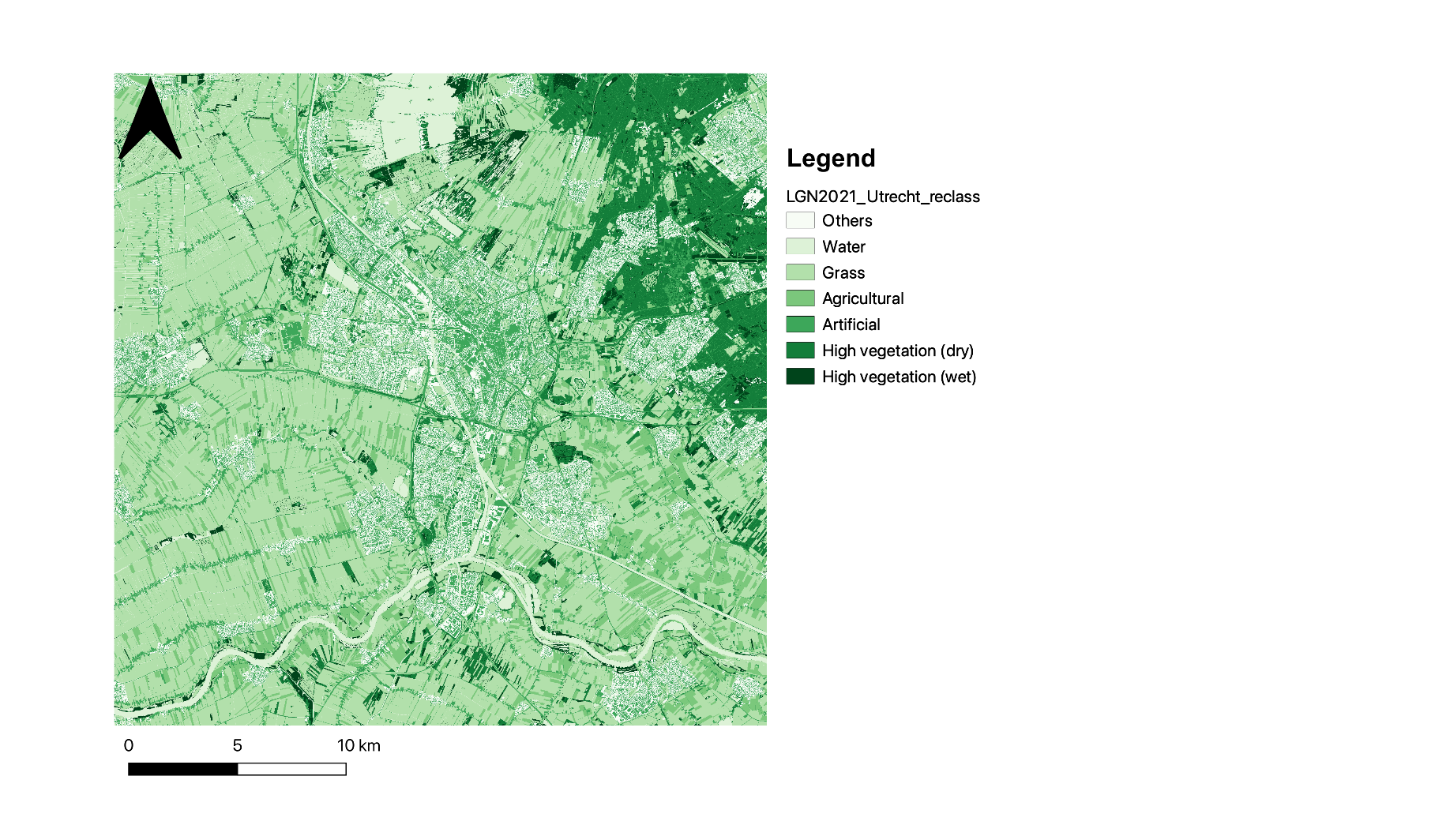

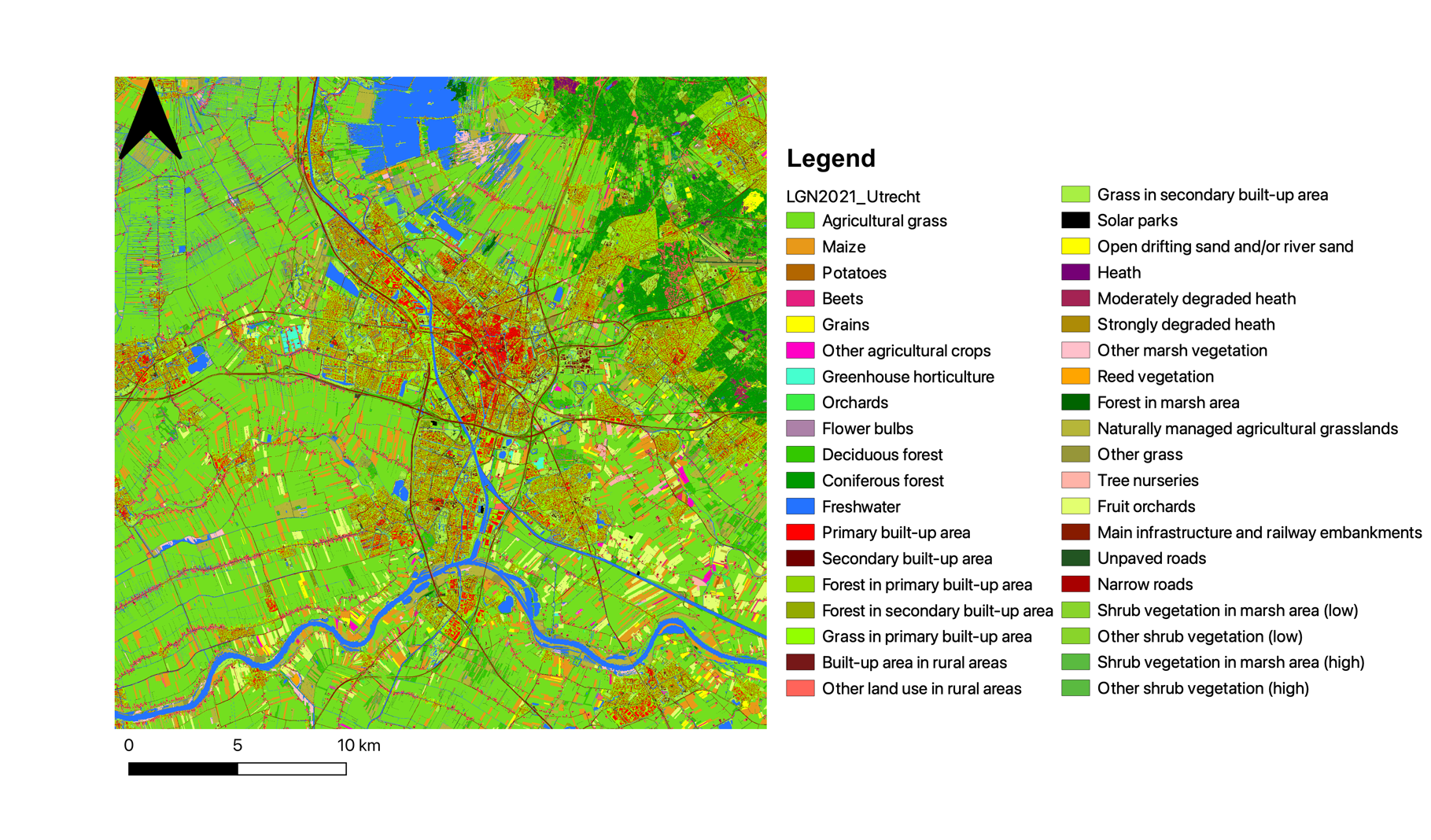
**Figure S2:** LGN2021 5x5 metres land cover map (CC BY-SA 4.0 Wageningen Environmental Research).

**Figure S3:** Reclassification of LGN2021 map into seven new classed based upon adult mosquito habitat suitability (for more details on the classification see Table S1).


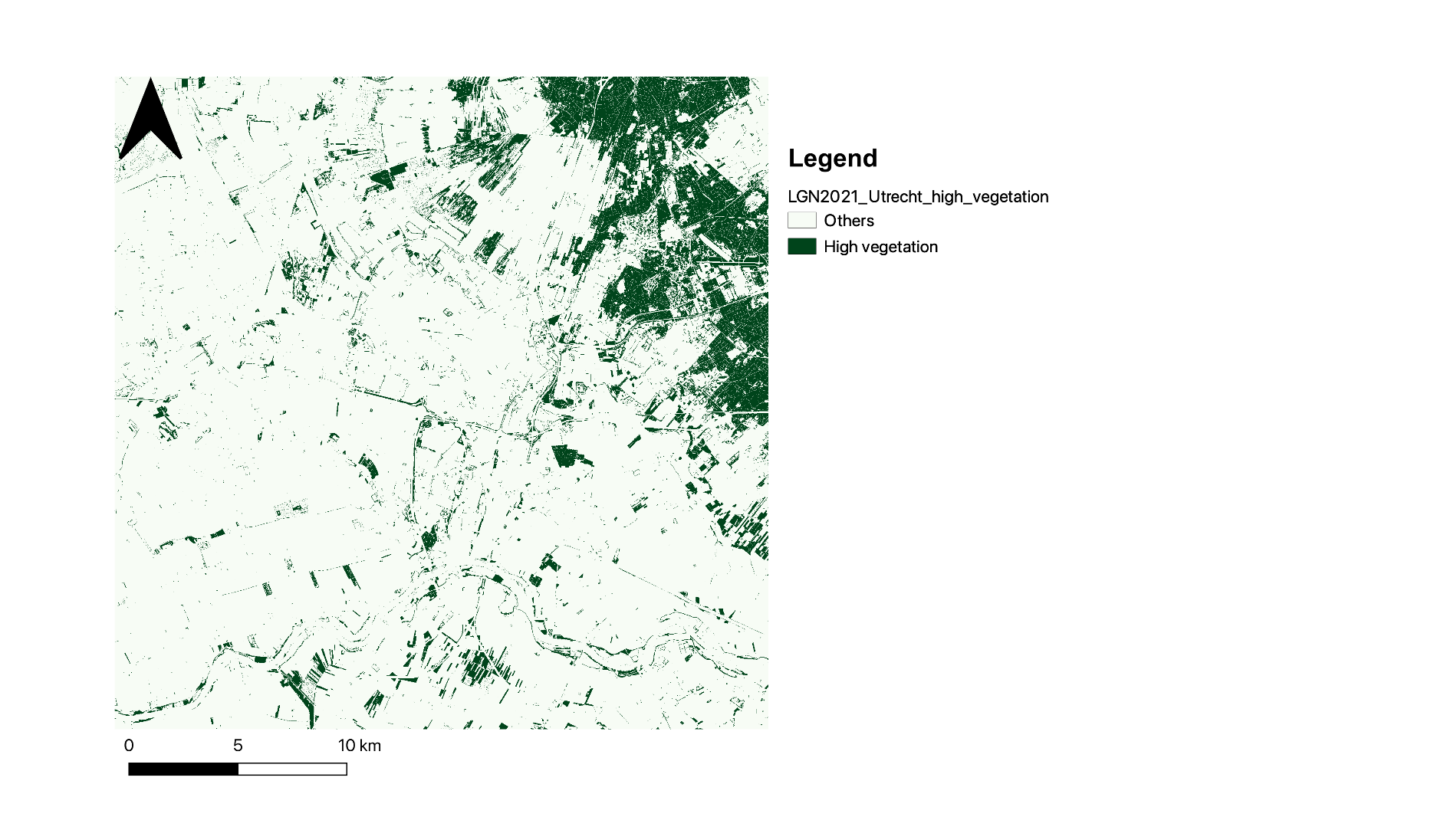
**Figure S4:** Reclassification of landscape suitability map into high vegetation and others.

**
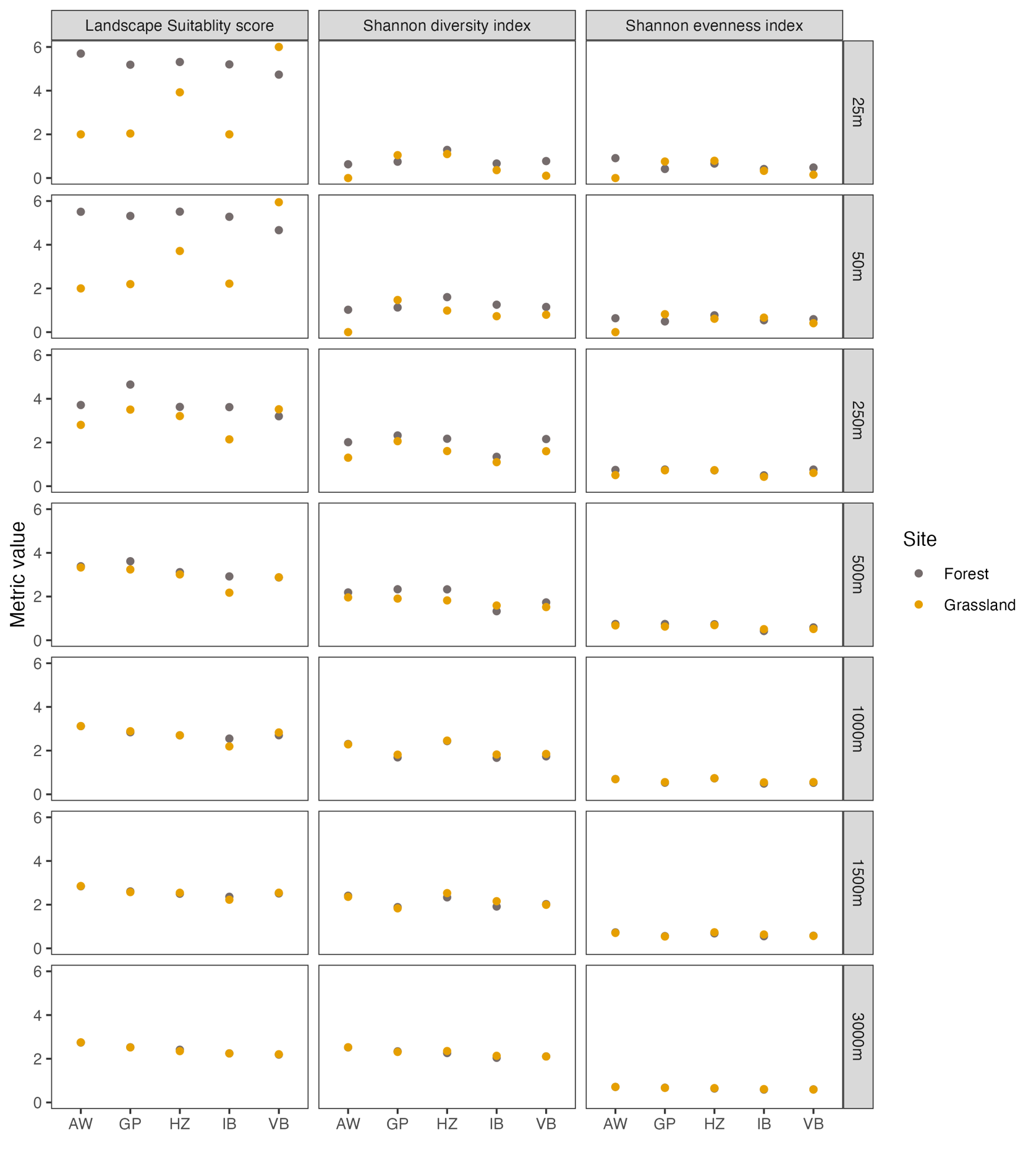
Figure S5:** Landscape metrics for each of the five locations and per forest patch and grassland site within a 25, 50, 250, 500, 1000, 1500 and 3000 metres buffer from the centroid of the site.


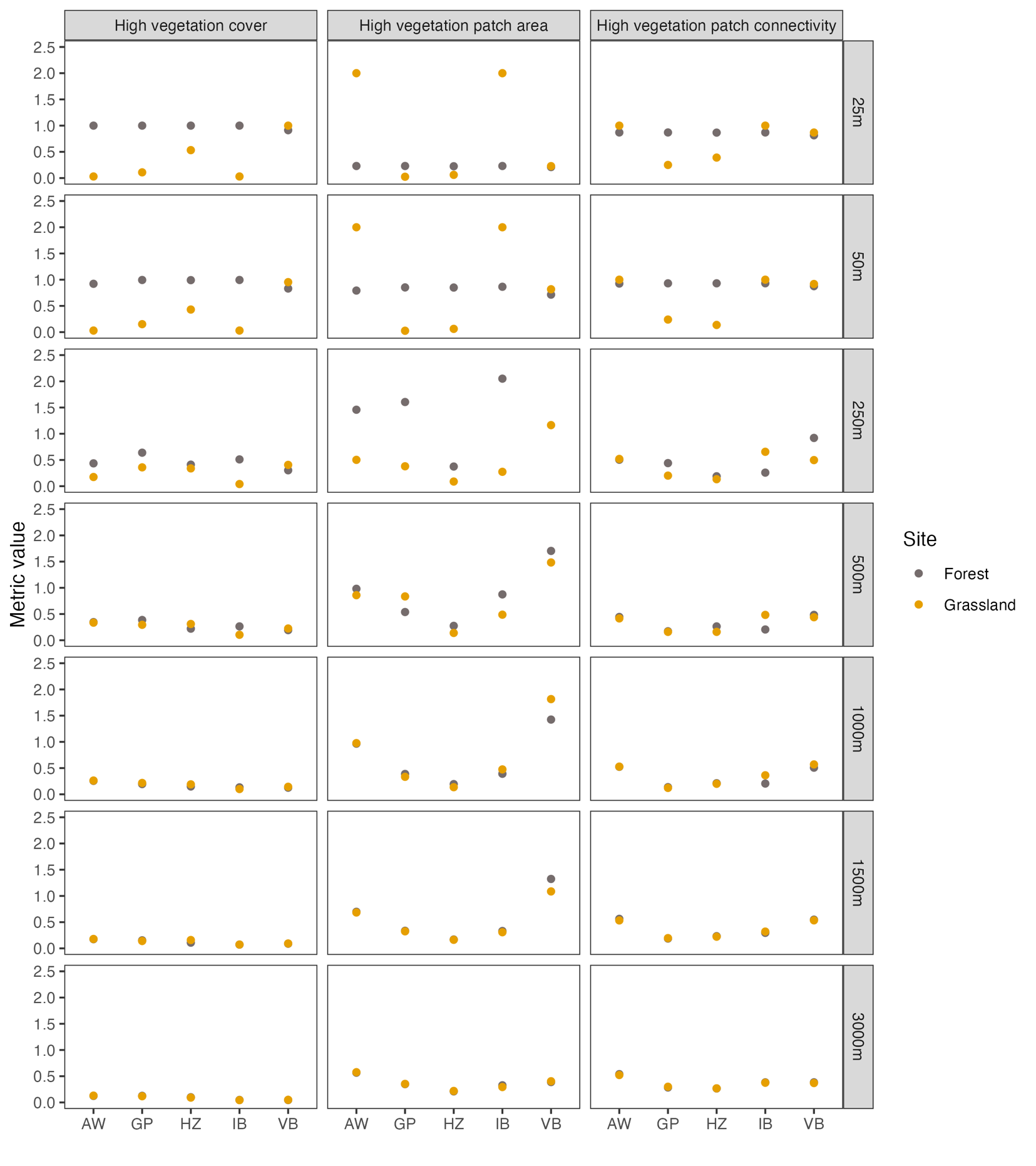
**Figure S6:** Landscape metrics for each of the five locations and per forest patch and grassland site within a 25, 50, 250, 500, 1000, 1500 and 3000 metres buffer from the centroid of the site.

**
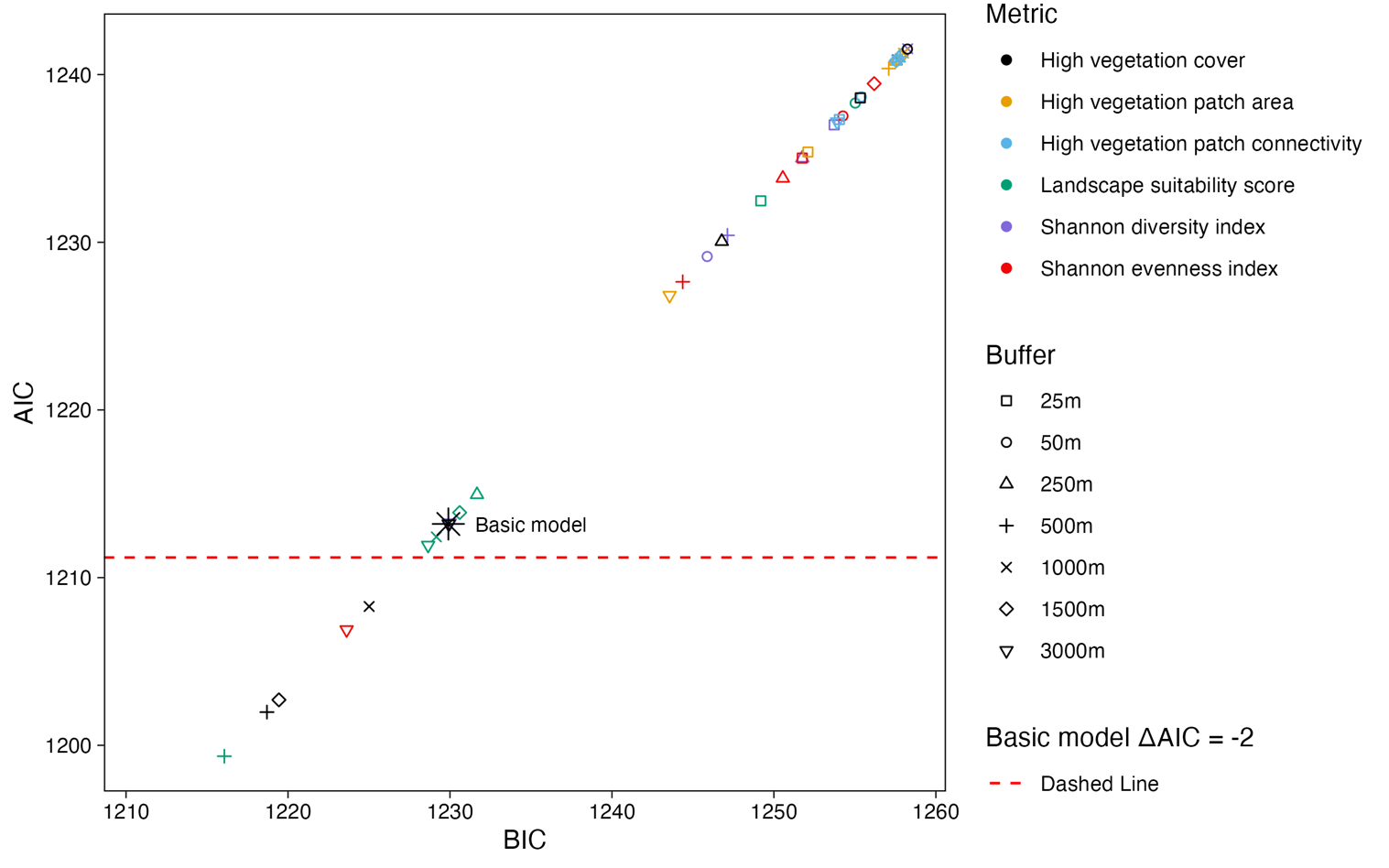
Figure S7:** Model performance to explain spatial pattern in mosquito abundance based on Akaike Information Criterion (AIC) and Bayesian Information Criterion (BIC) values. The basic model examines site (forest vs. grassland), location and their interaction. Subsequent models replace location with a landscape related for seven different buffer sizes. Models below the dashed red line have ΔAIC values of at least -2, indicating improved variance explanation compared to the basic model.

**Table S1:** LGN2021 5x5 metres land cover map (CC BY-SA 4.0 Wageningen Environmental Research). All 51 land cover classes were reclassified into seven new classed based upon adult mosquito habitat suitability, with 0 being not suitable and 6 highly suitable. These seven landscape suitability classes were then reclassified into high vegetation (1) and others (0).

| Original LGN2021 class | Landscape suitability class | High vegetation class |
| --- | --- | --- |
| Heath | High vegetation (wet)  Landscape suitability score 6 | High vegetation  (1) |
| Moderately degraded heath |  |  |
| Strongly degraded heath |  |  |
| Peat bog |  |  |
| Forest in peat bog area |  |  |
| Reed vegetation |  |  |
| Other marsh vegetation |  |  |
| Forest in marsh area |  |  |
| Other grass |  |  |
| Shrub vegetation in peat bog area (low) |  |  |
| Shrub vegetation in marsh area (low) |  |  |
| Shrub vegetation in peat bog area (high) |  |  |
| Shrub vegetation in marsh area (high) |  |  |
| Deciduous forest | High vegetation (dry)  Landscape suitability score 5 |  |
| Coniferous forest |  |  |
| Forest in primary built-up area |  |  |
| Forest in secondary built-up area |  |  |
| Other shrub vegetation (low) |  |  |
| Other shrub vegetation (high) |  |  |
| Greenhouse horticulture | Artificial  Landscape suitability score 4 | Others  (0) |
| Primary built-up area |  |  |
| Secondary built-up area |  |  |
| Roads and railways |  |  |
| Built-up area in rural areas |  |  |
| Other land use in rural areas |  |  |
| Solar parks |  |  |
| Main infrastructure and railway embankments |  |  |
| Unpaved roads |  |  |
| Narrow roads |  |  |
| Maize | Agricultural  Landscape suitability score 3 |  |
| Potatoes |  |  |
| Beets |  |  |
| Grains |  |  |
| Other agricultural crops |  |  |
| Orchards |  |  |
| Flower bulbs |  |  |
| Tree nurseries |  |  |
| Fruit orchards |  |  |
| Agricultural grass | Grass  Landscape suitability score 2 |  |
| Grass in secondary built-up area |  |  |
| Naturally managed agricultural grasslands |  |  |
| Grass in coastal areas |  |  |
| Freshwater | Freshwater  Landscape suitability score 1 |  |
| Saltwater | Others  Landscape suitability score 0 |  |
| Grass in primary built-up area |  |  |
| Bare ground in built-up area |  |  |
| Salt marshes |  |  |
| Open sand in coastal areas |  |  |
| Dunes with low vegetation |  |  |
| Dunes with high vegetation |  |  |
| Dune heath |  |  |
| Open drifting sand and/or river sand |  |  |

**Table S2:** Summary of ANOVA tests results. These models evaluate the differences in the *Shannon diversity index* (shdi) metric for site (forest vs. grassland) and location for each of the seven buffer distances. Model names are abbreviated using the metric and buffer distance.

| Model | Contrast | Estimate | SE | Df | t-ratio | P-value | Significance |
| --- | --- | --- | --- | --- | --- | --- | --- |
| shdi_25 | Forest - Grassland | 0.300 | 0.261 | 8 | 1.147 | 0.285 |  |
|  | AW - GP | -0.583 | 0.327 | 5 | -1.783 | 0.469 |  |
|  | AW - HZ | -0.877 | 0.327 | 5 | -2.684 | 0.188 |  |
|  | AW - IB | -0.199 | 0.327 | 5 | -0.609 | 0.967 |  |
|  | AW - VB | -0.126 | 0.327 | 5 | -0.385 | 0.994 |  |
|  | GP - HZ | -0.294 | 0.327 | 5 | -0.901 | 0.886 |  |
|  | GP - IB | 0.384 | 0.327 | 5 | 1.175 | 0.766 |  |
|  | GP - VB | 0.457 | 0.327 | 5 | 1.399 | 0.654 |  |
|  | HZ - IB | 0.678 | 0.327 | 5 | 2.075 | 0.353 |  |
|  | HZ - VB | 0.751 | 0.327 | 5 | 2.299 | 0.281 |  |
|  | IB - VB | 0.073 | 0.327 | 5 | 0.224 | 0.999 |  |
| shdi_50 | Forest - Grassland | 0.436 | 0.258 | 8 | 1.693 | 0.129 |  |
|  | AW - GP | -0.787 | 0.441 | 5 | -1.783 | 0.469 |  |
|  | AW - HZ | -0.782 | 0.441 | 5 | -1.772 | 0.474 |  |
|  | AW - IB | -0.480 | 0.441 | 5 | -1.088 | 0.807 |  |
|  | AW - VB | -0.462 | 0.441 | 5 | -1.046 | 0.826 |  |
|  | GP - HZ | 0.005 | 0.441 | 5 | 0.012 | 1 |  |
|  | GP - IB | 0.307 | 0.441 | 5 | 0.696 | 0.949 |  |
|  | GP - VB | 0.325 | 0.441 | 5 | 0.737 | 0.938 |  |
|  | HZ - IB | 0.302 | 0.441 | 5 | 0.684 | 0.952 |  |
|  | HZ - VB | 0.320 | 0.441 | 5 | 0.726 | 0.941 |  |
|  | IB - VB | 0.018 | 0.441 | 5 | 0.041 | 1 |  |
| shdi_250 | Forest - Grassland | 0.467 | 0.236 | 8 | 1.980 | < 0.1 | . |
|  | AW - GP | -0.533 | 0.355 | 5 | -1.502 | 0.602 |  |
|  | AW - HZ | -0.231 | 0.355 | 5 | -0.652 | 0.959 |  |
|  | AW - IB | 0.435 | 0.355 | 5 | 1.227 | 0.74 |  |
|  | AW - VB | -0.221 | 0.355 | 5 | -0.622 | 0.965 |  |
|  | GP - HZ | 0.302 | 0.355 | 5 | 0.850 | 0.903 |  |
|  | GP - IB | 0.968 | 0.355 | 5 | 2.729 | 0.179 |  |
|  | GP - VB | 0.312 | 0.355 | 5 | 0.880 | 0.893 |  |
|  | HZ - IB | 0.666 | 0.355 | 5 | 1.878 | 0.428 |  |
|  | HZ - VB | 0.010 | 0.355 | 5 | 0.029 | 1 |  |
|  | IB - VB | -0.656 | 0.355 | 5 | -1.849 | 0.44 |  |
| shdi_500 | Forest - Grassland | 0.222 | 0.215 | 8 | 1.032 | 0.332 |  |
|  | AW - GP | -0.045 | 0.245 | 5 | -0.185 | 1 |  |
|  | AW - HZ | -0.002 | 0.245 | 5 | -0.007 | 1 |  |
|  | AW - IB | 0.615 | 0.245 | 5 | 2.505 | 0.227 |  |
|  | AW - VB | 0.449 | 0.245 | 5 | 1.832 | 0.448 |  |
|  | GP - HZ | 0.044 | 0.245 | 5 | 0.178 | 1 |  |
|  | GP - IB | 0.660 | 0.245 | 5 | 2.690 | 0.187 |  |
|  | GP - VB | 0.495 | 0.245 | 5 | 2.017 | 0.374 |  |
|  | HZ - IB | 0.616 | 0.245 | 5 | 2.512 | 0.225 |  |
|  | HZ - VB | 0.451 | 0.245 | 5 | 1.839 | 0.445 |  |
|  | IB - VB | -0.165 | 0.245 | 5 | -0.673 | 0.954 |  |
| shdi_1000 | Forest - Grassland | -0.078 | 0.215 | 8 | -0.366 | 0.724 |  |
|  | AW - GP | 0.541 | 0.072 | 5 | 7.538 | < 0.01 | ** |
|  | AW - HZ | -0.154 | 0.072 | 5 | -2.146 | 0.328 |  |
|  | AW - IB | 0.547 | 0.072 | 5 | 7.621 | < 0.01 | ** |
|  | AW - VB | 0.502 | 0.072 | 5 | 6.988 | < 0.01 | ** |
|  | GP - HZ | -0.695 | 0.072 | 5 | -9.684 | < 0.01 | ** |
|  | GP - IB | 0.006 | 0.072 | 5 | 0.083 | 1 |  |
|  | GP - VB | -0.039 | 0.072 | 5 | -0.550 | 0.977 |  |
|  | HZ - IB | 0.701 | 0.072 | 5 | 9.767 | < 0.01 | ** |
|  | HZ - VB | 0.656 | 0.072 | 5 | 9.134 | < 0.01 | ** |
|  | IB - VB | -0.045 | 0.072 | 5 | -0.633 | 0.963 |  |
| shdi_1500 | Forest - Grassland | -0.060 | 0.165 | 8 | -0.364 | 0.725 |  |
|  | AW - GP | 0.523 | 0.102 | 5 | 5.142 | < 0.05 | * |
|  | AW - HZ | -0.048 | 0.102 | 5 | -0.472 | 0.987 |  |
|  | AW - IB | 0.347 | 0.102 | 5 | 3.411 | < 0.1 | . |
|  | AW - VB | 0.374 | 0.102 | 5 | 3.678 | < 0.1 | . |
|  | GP - HZ | -0.571 | 0.102 | 5 | -5.614 | < 0.05 | * |
|  | GP - IB | -0.176 | 0.102 | 5 | -1.731 | 0.492 |  |
|  | GP - VB | -0.149 | 0.102 | 5 | -1.464 | 0.621 |  |
|  | HZ - IB | 0.395 | 0.102 | 5 | 3.883 | < 0.1 | . |
|  | HZ - VB | 0.422 | 0.102 | 5 | 4.150 | < 0.05 | * |
|  | IB - VB | 0.027 | 0.102 | 5 | 0.267 | 0.998 |  |
| shdi_3000 | Forest - Grassland | -0.032 | 0.115 | 8 | -0.277 | 0.788 |  |
|  | AW - GP | 0.198 | 0.041 | 5 | 4.799 | < 0.05 | * |
|  | AW - HZ | 0.217 | 0.041 | 5 | 5.262 | < 0.05 | * |
|  | AW - IB | 0.435 | 0.041 | 5 | 10.529 | < 0.001 | *** |
|  | AW - VB | 0.418 | 0.041 | 5 | 10.129 | < 0.001 | *** |
|  | GP - HZ | 0.019 | 0.041 | 5 | 0.464 | 0.988 |  |
|  | GP - IB | 0.237 | 0.041 | 5 | 5.730 | < 0.05 | * |
|  | GP - VB | 0.220 | 0.041 | 5 | 5.330 | < 0.05 | * |
|  | HZ - IB | 0.218 | 0.041 | 5 | 5.266 | < 0.05 | * |
|  | HZ - VB | 0.201 | 0.041 | 5 | 4.867 | < 0.05 | * |
|  | IB - VB | -0.017 | 0.041 | 5 | -0.400 | 0.993 |  |

**Table S3:** Summary of ANOVA tests results. These models evaluate the differences in the *Shannon evenness index* (shei) metric for site (forest vs. grassland) and location for each of the seven buffer distances. Model names are abbreviated using the metric and buffer distance.

| Model | Contrast | Estimate | SE | Df | t-ratio | P-value | Significance |
| --- | --- | --- | --- | --- | --- | --- | --- |
| shei_25 | Forest - Grassland | 0.171 | 0.185 | 8 | 0.926 | 0.382 |  |
|  | AW - GP | -0.132 | 0.328 | 5 | -0.401 | 0.993 |  |
|  | AW - HZ | -0.271 | 0.328 | 5 | -0.827 | 0.911 |  |
|  | AW - IB | 0.083 | 0.328 | 5 | 0.254 | 0.999 |  |
|  | AW - VB | 0.138 | 0.328 | 5 | 0.420 | 0.991 |  |
|  | GP - HZ | -0.140 | 0.328 | 5 | -0.426 | 0.991 |  |
|  | GP - IB | 0.215 | 0.328 | 5 | 0.655 | 0.958 |  |
|  | GP - VB | 0.269 | 0.328 | 5 | 0.821 | 0.913 |  |
|  | HZ - IB | 0.355 | 0.328 | 5 | 1.081 | 0.81 |  |
|  | HZ - VB | 0.409 | 0.328 | 5 | 1.247 | 0.73 |  |
|  | IB - VB | 0.055 | 0.328 | 5 | 0.166 | 1 |  |
| shei_50 | Forest - Grassland | 0.106 | 0.149 | 8 | 0.707 | 0.499 |  |
|  | AW - GP | -0.337 | 0.242 | 5 | -1.393 | 0.657 |  |
|  | AW - HZ | -0.374 | 0.242 | 5 | -1.543 | 0.581 |  |
|  | AW - IB | -0.286 | 0.242 | 5 | -1.182 | 0.762 |  |
|  | AW - VB | -0.182 | 0.242 | 5 | -0.753 | 0.934 |  |
|  | GP - HZ | -0.036 | 0.242 | 5 | -0.150 | 1 |  |
|  | GP - IB | 0.051 | 0.242 | 5 | 0.211 | 0.999 |  |
|  | GP - VB | 0.155 | 0.242 | 5 | 0.640 | 0.962 |  |
|  | HZ - IB | 0.087 | 0.242 | 5 | 0.361 | 0.995 |  |
|  | HZ - VB | 0.191 | 0.242 | 5 | 0.789 | 0.923 |  |
|  | IB - VB | 0.104 | 0.242 | 5 | 0.429 | 0.991 |  |
| shei_250 | Forest - Grassland | 0.097 | 0.078 | 8 | 1.242 | 0.249 |  |
|  | AW - GP | -0.119 | 0.092 | 5 | -1.291 | 0.708 |  |
|  | AW - HZ | -0.102 | 0.092 | 5 | -1.110 | 0.797 |  |
|  | AW - IB | 0.163 | 0.092 | 5 | 1.763 | 0.477 |  |
|  | AW - VB | -0.058 | 0.092 | 5 | -0.631 | 0.963 |  |
|  | GP - HZ | 0.017 | 0.092 | 5 | 0.181 | 1 |  |
|  | GP - IB | 0.282 | 0.092 | 5 | 3.054 | 0.128 |  |
|  | GP - VB | 0.061 | 0.092 | 5 | 0.660 | 0.957 |  |
|  | HZ - IB | 0.265 | 0.092 | 5 | 2.873 | 0.154 |  |
|  | HZ - VB | 0.044 | 0.092 | 5 | 0.479 | 0.986 |  |
|  | IB - VB | -0.221 | 0.092 | 5 | -2.394 | 0.254 |  |
| shei_500 | Forest - Grassland | 0.043 | 0.074 | 8 | 0.576 | 0.581 |  |
|  | AW - GP | 0.026 | 0.057 | 5 | 0.453 | 0.989 |  |
|  | AW - HZ | -0.001 | 0.057 | 5 | -0.020 | 1 |  |
|  | AW - IB | 0.245 | 0.057 | 5 | 4.338 | < 0.05 | * |
|  | AW - VB | 0.159 | 0.057 | 5 | 2.811 | 0.165 |  |
|  | GP - HZ | -0.027 | 0.057 | 5 | -0.473 | 0.987 |  |
|  | GP - IB | 0.220 | 0.057 | 5 | 3.885 | < 0.1 | . |
|  | GP - VB | 0.133 | 0.057 | 5 | 2.358 | 0.264 |  |
|  | HZ - IB | 0.246 | 0.057 | 5 | 4.358 | < 0.05 | * |
|  | HZ - VB | 0.160 | 0.057 | 5 | 2.831 | 0.161 |  |
|  | IB - VB | -0.086 | 0.057 | 5 | -1.527 | 0.589 |  |
| shei_1000 | Forest - Grassland | -0.023 | 0.064 | 8 | -0.365 | 0.725 |  |
|  | AW - GP | 0.151 | 0.022 | 5 | 6.828 | < 0.01 | ** |
|  | AW - HZ | -0.039 | 0.022 | 5 | -1.741 | 0.487 |  |
|  | AW - IB | 0.177 | 0.022 | 5 | 7.972 | < 0.01 | ** |
|  | AW - VB | 0.152 | 0.022 | 5 | 6.860 | < 0.01 | ** |
|  | GP - HZ | -0.190 | 0.022 | 5 | -8.570 | < 0.01 | ** |
|  | GP - IB | 0.025 | 0.022 | 5 | 1.143 | 0.781 |  |
|  | GP - VB | 0.001 | 0.022 | 5 | 0.032 | 1 |  |
|  | HZ - IB | 0.216 | 0.022 | 5 | 9.713 | < 0.01 | ** |
|  | HZ - VB | 0.191 | 0.022 | 5 | 8.601 | < 0.01 | ** |
|  | IB - VB | -0.025 | 0.022 | 5 | -1.112 | 0.796 |  |
| shei_1500 | Forest - Grassland | -0.016 | 0.052 | 8 | -0.302 | 0.77 |  |
|  | AW - GP | 0.167 | 0.031 | 5 | 5.470 | < 0.05 | * |
|  | AW - HZ | 0.008 | 0.031 | 5 | 0.260 | 0.999 |  |
|  | AW - IB | 0.123 | 0.031 | 5 | 4.041 | < 0.05 | * |
|  | AW - VB | 0.145 | 0.031 | 5 | 4.743 | < 0.05 | * |
|  | GP - HZ | -0.159 | 0.031 | 5 | -5.210 | < 0.05 | * |
|  | GP - IB | -0.044 | 0.031 | 5 | -1.429 | 0.638 |  |
|  | GP - VB | -0.022 | 0.031 | 5 | -0.727 | 0.941 |  |
|  | HZ - IB | 0.115 | 0.031 | 5 | 3.781 | < 0.1 | . |
|  | HZ - VB | 0.137 | 0.031 | 5 | 4.482 | < 0.05 | * |
|  | IB - VB | 0.021 | 0.031 | 5 | 0.702 | 0.947 |  |
| shei_3000 | Forest - Grassland | -0.006 | 0.031 | 8 | -0.192 | 0.853 |  |
|  | AW - GP | 0.039 | 0.010 | 5 | 4.031 | < 0.05 | * |
|  | AW - HZ | 0.064 | 0.010 | 5 | 6.614 | < 0.01 | ** |
|  | AW - IB | 0.110 | 0.010 | 5 | 11.399 | < 0.001 | *** |
|  | AW - VB | 0.115 | 0.010 | 5 | 11.959 | < 0.001 | *** |
|  | GP - HZ | 0.025 | 0.010 | 5 | 2.583 | 0.209 |  |
|  | GP - IB | 0.071 | 0.010 | 5 | 7.368 | < 0.01 | ** |
|  | GP - VB | 0.076 | 0.010 | 5 | 7.928 | < 0.01 | ** |
|  | HZ - IB | 0.046 | 0.010 | 5 | 4.785 | < 0.05 | * |
|  | HZ - VB | 0.052 | 0.010 | 5 | 5.345 | < 0.05 | * |
|  | IB - VB | 0.005 | 0.010 | 5 | 0.560 | 0.976 |  |

**Table S4:** Summary of ANOVA tests results. These models evaluate the differences in the *landscape suitability score* (per_sl) metric for site (forest vs. grassland) and location for each of the seven buffer distances. Model names are abbreviated using the metric and buffer distance.

| Model | Contrast | Estimate | SE | Df | t-ratio | P-value | Significance |
| --- | --- | --- | --- | --- | --- | --- | --- |
| per_sl_25 | Forest - Grassland | 2.034 | 0.808 | 8 | 2.516 | < 0.05 | * |
|  | AW - GP | 0.236 | 1.933 | 5 | 0.122 | 1 |  |
|  | AW - HZ | -0.770 | 1.933 | 5 | -0.398 | 0.993 |  |
|  | AW - IB | 0.247 | 1.933 | 5 | 0.128 | 1 |  |
|  | AW - VB | -1.519 | 1.933 | 5 | -0.786 | 0.924 |  |
|  | GP - HZ | -1.005 | 1.933 | 5 | -0.520 | 0.981 |  |
|  | GP - IB | 0.011 | 1.933 | 5 | 0.006 | 1 |  |
|  | GP - VB | -1.755 | 1.933 | 5 | -0.908 | 0.883 |  |
|  | HZ - IB | 1.017 | 1.933 | 5 | 0.526 | 0.981 |  |
|  | HZ - VB | -0.749 | 1.933 | 5 | -0.388 | 0.994 |  |
|  | IB - VB | -1.766 | 1.933 | 5 | -0.913 | 0.881 |  |
| per_sl_50 | Forest - Grassland | 2.042 | 0.765 | 8 | 2.670 | < 0.05 | * |
|  | AW - GP | -0.002 | 1.905 | 5 | -0.001 | 1 |  |
|  | AW - HZ | -0.859 | 1.905 | 5 | -0.451 | 0.989 |  |
|  | AW - IB | 0.006 | 1.905 | 5 | 0.003 | 1 |  |
|  | AW - VB | -1.551 | 1.905 | 5 | -0.814 | 0.915 |  |
|  | GP - HZ | -0.857 | 1.905 | 5 | -0.450 | 0.989 |  |
|  | GP - IB | 0.008 | 1.905 | 5 | 0.004 | 1 |  |
|  | GP - VB | -1.549 | 1.905 | 5 | -0.813 | 0.916 |  |
|  | HZ - IB | 0.865 | 1.905 | 5 | 0.454 | 0.989 |  |
|  | HZ - VB | -0.692 | 1.905 | 5 | -0.363 | 0.995 |  |
|  | IB - VB | -1.557 | 1.905 | 5 | -0.817 | 0.914 |  |
| per_sl_250 | Forest - Grassland | 0.726 | 0.352 | 8 | 2.062 | < 0.1 | . |
|  | AW - GP | -0.818 | 0.678 | 5 | -1.207 | 0.75 |  |
|  | AW - HZ | -0.160 | 0.678 | 5 | -0.235 | 0.999 |  |
|  | AW - IB | 0.379 | 0.678 | 5 | 0.560 | 0.976 |  |
|  | AW - VB | -0.101 | 0.678 | 5 | -0.148 | 1 |  |
|  | GP - HZ | 0.659 | 0.678 | 5 | 0.971 | 0.858 |  |
|  | GP - IB | 1.198 | 0.678 | 5 | 1.767 | 0.476 |  |
|  | GP - VB | 0.718 | 0.678 | 5 | 1.059 | 0.82 |  |
|  | HZ - IB | 0.539 | 0.678 | 5 | 0.795 | 0.922 |  |
|  | HZ - VB | 0.059 | 0.678 | 5 | 0.087 | 1 |  |
|  | IB - VB | -0.480 | 0.678 | 5 | -0.708 | 0.946 |  |
| per_sl_500 | Forest - Grassland | 0.255 | 0.248 | 8 | 1.027 | 0.335 |  |
|  | AW - GP | -0.070 | 0.267 | 5 | -0.264 | 0.999 |  |
|  | AW - HZ | 0.293 | 0.267 | 5 | 1.098 | 0.802 |  |
|  | AW - IB | 0.809 | 0.267 | 5 | 3.032 | 0.131 |  |
|  | AW - VB | 0.480 | 0.267 | 5 | 1.799 | 0.462 |  |
|  | GP - HZ | 0.363 | 0.267 | 5 | 1.362 | 0.672 |  |
|  | GP - IB | 0.879 | 0.267 | 5 | 3.296 | 0.1 |  |
|  | GP - VB | 0.550 | 0.267 | 5 | 2.063 | 0.357 |  |
|  | HZ - IB | 0.516 | 0.267 | 5 | 1.934 | 0.405 |  |
|  | HZ - VB | 0.187 | 0.267 | 5 | 0.700 | 0.948 |  |
|  | IB - VB | -0.329 | 0.267 | 5 | -1.234 | 0.737 |  |
| per_sl_1000 | Forest - Grassland | 0.036 | 0.182 | 8 | 0.196 | 0.849 |  |
|  | AW - GP | 0.258 | 0.121 | 5 | 2.134 | 0.332 |  |
|  | AW - HZ | 0.421 | 0.121 | 5 | 3.481 | < 0.1 | . |
|  | AW - IB | 0.750 | 0.121 | 5 | 6.199 | < 0.01 | ** |
|  | AW - VB | 0.355 | 0.121 | 5 | 2.936 | 0.145 |  |
|  | GP - HZ | 0.163 | 0.121 | 5 | 1.346 | 0.68 |  |
|  | GP - IB | 0.492 | 0.121 | 5 | 4.065 | < 0.05 | * |
|  | GP - VB | 0.097 | 0.121 | 5 | 0.802 | 0.92 |  |
|  | HZ - IB | 0.329 | 0.121 | 5 | 2.719 | 0.181 |  |
|  | HZ - VB | -0.066 | 0.121 | 5 | -0.545 | 0.978 |  |
|  | IB - VB | -0.395 | 0.121 | 5 | -3.264 | 0.104 |  |
| per_sl_1500 | Forest - Grassland | 0.016 | 0.127 | 8 | 0.127 | 0.902 |  |
|  | AW - GP | 0.257 | 0.047 | 5 | 5.439 | < 0.05 | * |
|  | AW - HZ | 0.326 | 0.047 | 5 | 6.884 | < 0.01 | ** |
|  | AW - IB | 0.553 | 0.047 | 5 | 11.704 | < 0.001 | *** |
|  | AW - VB | 0.318 | 0.047 | 5 | 6.723 | < 0.01 | ** |
|  | GP - HZ | 0.068 | 0.047 | 5 | 1.445 | 0.63 |  |
|  | GP - IB | 0.296 | 0.047 | 5 | 6.266 | < 0.01 | ** |
|  | GP - VB | 0.061 | 0.047 | 5 | 1.284 | 0.712 |  |
|  | HZ - IB | 0.228 | 0.047 | 5 | 4.820 | < 0.05 | * |
|  | HZ - VB | -0.008 | 0.047 | 5 | -0.161 | 1 |  |
|  | IB - VB | -0.236 | 0.047 | 5 | -4.981 | < 0.05 | * |
| per_sl_3000 | Forest - Grassland | 0.009 | 0.141 | 8 | 0.066 | 0.949 |  |
|  | AW - GP | 0.222 | 0.021 | 5 | 10.455 | < 0.001 | *** |
|  | AW - HZ | 0.365 | 0.021 | 5 | 17.175 | < 0.001 | *** |
|  | AW - IB | 0.501 | 0.021 | 5 | 23.555 | < 0.001 | *** |
|  | AW - VB | 0.549 | 0.021 | 5 | 25.799 | < 0.001 | *** |
|  | GP - HZ | 0.143 | 0.021 | 5 | 6.721 | < 0.01 | ** |
|  | GP - IB | 0.279 | 0.021 | 5 | 13.100 | < 0.001 | *** |
|  | GP - VB | 0.326 | 0.021 | 5 | 15.344 | < 0.001 | *** |
|  | HZ - IB | 0.136 | 0.021 | 5 | 6.380 | < 0.01 | ** |
|  | HZ - VB | 0.183 | 0.021 | 5 | 8.624 | < 0.01 | ** |
|  | IB - VB | 0.048 | 0.021 | 5 | 2.244 | 0.297 |  |

**Table S5:** Summary of ANOVA tests results. These models evaluate the differences in the *high vegetation cover* (pland_high) metric for site (forest vs. grassland) and location for each of the seven buffer distances. Model names are abbreviated using the metric and buffer distance.

| Model | Contrast | Estimate | SE | Df | t-ratio | P-value | Significance |
| --- | --- | --- | --- | --- | --- | --- | --- |
| pland_high_25 | Forest - Grassland | 64.235 | 19.032 | 8 | 3.375 | < 0.01 | ** |
|  | AW - GP | -3.935 | 53.872 | 5 | -0.073 | 1 |  |
|  | AW - HZ | -25.130 | 53.872 | 5 | -0.466 | 0.987 |  |
|  | AW - IB | 0.000 | 53.872 | 5 | 0.000 | 1 |  |
|  | AW - VB | -44.152 | 53.872 | 5 | -0.820 | 0.914 |  |
|  | GP - HZ | -21.196 | 53.872 | 5 | -0.393 | 0.993 |  |
|  | GP - IB | 3.935 | 53.872 | 5 | 0.073 | 1 |  |
|  | GP - VB | -40.217 | 53.872 | 5 | -0.747 | 0.936 |  |
|  | HZ - IB | 25.130 | 53.872 | 5 | 0.466 | 0.987 |  |
|  | HZ - VB | -19.022 | 53.872 | 5 | -0.353 | 0.996 |  |
|  | IB - VB | -44.152 | 53.872 | 5 | -0.820 | 0.914 |  |
| pland_high_50 | Forest - Grassland | 62.740 | 17.753 | 8 | 3.534 | < 0.01 | ** |
|  | AW - GP | -9.713 | 52.571 | 5 | -0.185 | 1 |  |
|  | AW - HZ | -23.519 | 52.571 | 5 | -0.447 | 0.989 |  |
|  | AW - IB | -3.637 | 52.571 | 5 | -0.069 | 1 |  |
|  | AW - VB | -41.662 | 52.571 | 5 | -0.792 | 0.922 |  |
|  | GP - HZ | -13.806 | 52.571 | 5 | -0.263 | 0.999 |  |
|  | GP - IB | 6.076 | 52.571 | 5 | 0.116 | 1 |  |
|  | GP - VB | -31.949 | 52.571 | 5 | -0.608 | 0.968 |  |
|  | HZ - IB | 19.882 | 52.571 | 5 | 0.378 | 0.994 |  |
|  | HZ - VB | -18.143 | 52.571 | 5 | -0.345 | 0.996 |  |
|  | IB - VB | -38.025 | 52.571 | 5 | -0.723 | 0.942 |  |
| pland_high_250 | Forest - Grassland | 19.578 | 8.799 | 8 | 2.225 | < 0.1 | . |
|  | AW - GP | -19.380 | 19.564 | 5 | -0.991 | 0.85 |  |
|  | AW - HZ | -6.962 | 19.564 | 5 | -0.356 | 0.995 |  |
|  | AW - IB | 2.940 | 19.564 | 5 | 0.150 | 1 |  |
|  | AW - VB | -4.947 | 19.564 | 5 | -0.253 | 0.999 |  |
|  | GP - HZ | 12.418 | 19.564 | 5 | 0.635 | 0.962 |  |
|  | GP - IB | 22.319 | 19.564 | 5 | 1.141 | 0.782 |  |
|  | GP - VB | 14.433 | 19.564 | 5 | 0.738 | 0.938 |  |
|  | HZ - IB | 9.902 | 19.564 | 5 | 0.506 | 0.983 |  |
|  | HZ - VB | 2.015 | 19.564 | 5 | 0.103 | 1 |  |
|  | IB - VB | -7.886 | 19.564 | 5 | -0.403 | 0.993 |  |
| pland_high_500 | Forest - Grassland | 2.826 | 5.548 | 8 | 0.509 | 0.624 |  |
|  | AW - GP | 0.032 | 6.547 | 5 | 0.005 | 1 |  |
|  | AW - HZ | 7.505 | 6.547 | 5 | 1.146 | 0.779 |  |
|  | AW - IB | 15.638 | 6.547 | 5 | 2.389 | 0.256 |  |
|  | AW - VB | 13.208 | 6.547 | 5 | 2.017 | 0.373 |  |
|  | GP - HZ | 7.473 | 6.547 | 5 | 1.141 | 0.782 |  |
|  | GP - IB | 15.606 | 6.547 | 5 | 2.384 | 0.257 |  |
|  | GP - VB | 13.176 | 6.547 | 5 | 2.012 | 0.375 |  |
|  | HZ - IB | 8.134 | 6.547 | 5 | 1.242 | 0.732 |  |
|  | HZ - VB | 5.703 | 6.547 | 5 | 0.871 | 0.896 |  |
|  | IB - VB | -2.431 | 6.547 | 5 | -0.371 | 0.995 |  |
| pland_high_1000 | Forest - Grassland | -1.061 | 3.723 | 8 | -0.285 | 0.783 |  |
|  | AW - GP | 5.379 | 1.854 | 5 | 2.901 | 0.15 |  |
|  | AW - HZ | 9.011 | 1.854 | 5 | 4.859 | < 0.05 | * |
|  | AW - IB | 14.310 | 1.854 | 5 | 7.717 | < 0.01 | ** |
|  | AW - VB | 12.474 | 1.854 | 5 | 6.727 | < 0.01 | ** |
|  | GP - HZ | 3.632 | 1.854 | 5 | 1.959 | 0.396 |  |
|  | GP - IB | 8.932 | 1.854 | 5 | 4.817 | < 0.05 | * |
|  | GP - VB | 7.095 | 1.854 | 5 | 3.826 | < 0.1 | . |
|  | HZ - IB | 5.300 | 1.854 | 5 | 2.858 | 0.157 |  |
|  | HZ - VB | 3.463 | 1.854 | 5 | 1.868 | 0.433 |  |
|  | IB - VB | -1.837 | 1.854 | 5 | -0.990 | 0.85 |  |
| pland_high_1500 | Forest - Grassland | -0.923 | 2.785 | 8 | -0.332 | 0.749 |  |
|  | AW - GP | 3.012 | 1.640 | 5 | 1.837 | 0.446 |  |
|  | AW - HZ | 4.327 | 1.640 | 5 | 2.639 | 0.197 |  |
|  | AW - IB | 10.485 | 1.640 | 5 | 6.395 | < 0.01 | ** |
|  | AW - VB | 8.575 | 1.640 | 5 | 5.230 | < 0.05 | * |
|  | GP - HZ | 1.316 | 1.640 | 5 | 0.803 | 0.919 |  |
|  | GP - IB | 7.474 | 1.640 | 5 | 4.558 | < 0.05 | * |
|  | GP - VB | 5.564 | 1.640 | 5 | 3.393 | < 0.1 | . |
|  | HZ - IB | 6.158 | 1.640 | 5 | 3.756 | < 0.1 | . |
|  | HZ - VB | 4.248 | 1.640 | 5 | 2.591 | 0.207 |  |
|  | IB - VB | -1.910 | 1.640 | 5 | -1.165 | 0.77 |  |
| pland_high_3000 | Forest - Grassland | 0.161 | 2.483 | 8 | 0.065 | 0.95 |  |
|  | AW - GP | 0.495 | 0.408 | 5 | 1.214 | 0.746 |  |
|  | AW - HZ | 3.144 | 0.408 | 5 | 7.712 | < 0.01 | ** |
|  | AW - IB | 8.115 | 0.408 | 5 | 19.905 | < 0.001 | *** |
|  | AW - VB | 7.918 | 0.408 | 5 | 19.422 | < 0.001 | *** |
|  | GP - HZ | 2.649 | 0.408 | 5 | 6.497 | < 0.01 | ** |
|  | GP - IB | 7.620 | 0.408 | 5 | 18.691 | < 0.001 | *** |
|  | GP - VB | 7.423 | 0.408 | 5 | 18.208 | < 0.001 | *** |
|  | HZ - IB | 4.971 | 0.408 | 5 | 12.193 | < 0.001 | *** |
|  | HZ - VB | 4.774 | 0.408 | 5 | 11.710 | < 0.001 | *** |
|  | IB - VB | -0.197 | 0.408 | 5 | -0.483 | 0.986 |  |

**Table S6:** Summary of ANOVA tests results. These models evaluate the differences in the *high vegetation patch area* (area_mn_high) metric for site (forest vs. grassland) and location for each of the seven buffer distances. Model names are abbreviated using the metric and buffer distance.

| Model | Contrast | Estimate | SE | Df | t-ratio | P-value | Significance |
| --- | --- | --- | --- | --- | --- | --- | --- |
| area_mn_high_25 | Forest - Grassland | -0.638 | 0.466 | 8 | -1.370 | 0.208 |  |
|  | AW - GP | 0.988 | 0.796 | 5 | 1.241 | 0.733 |  |
|  | AW - HZ | 0.972 | 0.796 | 5 | 1.221 | 0.743 |  |
|  | AW - IB | 0.000 | 0.796 | 5 | 0.000 | 1 |  |
|  | AW - VB | 0.896 | 0.796 | 5 | 1.126 | 0.789 |  |
|  | GP - HZ | -0.016 | 0.796 | 5 | -0.020 | 1 |  |
|  | GP - IB | -0.988 | 0.796 | 5 | -1.241 | 0.733 |  |
|  | GP - VB | -0.091 | 0.796 | 5 | -0.115 | 1 |  |
|  | HZ - IB | -0.972 | 0.796 | 5 | -1.221 | 0.743 |  |
|  | HZ - VB | -0.076 | 0.796 | 5 | -0.095 | 1 |  |
|  | IB - VB | 0.896 | 0.796 | 5 | 1.126 | 0.789 |  |
| area_mn_high_50 | Forest - Grassland | -0.166 | 0.440 | 8 | -0.377 | 0.716 |  |
|  | AW - GP | 0.957 | 0.637 | 5 | 1.502 | 0.602 |  |
|  | AW - HZ | 0.940 | 0.637 | 5 | 1.475 | 0.615 |  |
|  | AW - IB | -0.036 | 0.637 | 5 | -0.057 | 1 |  |
|  | AW - VB | 0.630 | 0.637 | 5 | 0.989 | 0.851 |  |
|  | GP - HZ | -0.017 | 0.637 | 5 | -0.026 | 1 |  |
|  | GP - IB | -0.993 | 0.637 | 5 | -1.559 | 0.574 |  |
|  | GP - VB | -0.327 | 0.637 | 5 | -0.513 | 0.982 |  |
|  | HZ - IB | -0.976 | 0.637 | 5 | -1.532 | 0.587 |  |
|  | HZ - VB | -0.310 | 0.637 | 5 | -0.487 | 0.985 |  |
|  | IB - VB | 0.666 | 0.637 | 5 | 1.046 | 0.826 |  |
| area_mn_high_250 | Forest - Grassland | 1.225 | 0.471 | 8 | 2.602 | < 0.05 | * |
|  | AW - GP | -0.012 | 0.959 | 5 | -0.013 | 1 |  |
|  | AW - HZ | 0.748 | 0.959 | 5 | 0.781 | 0.926 |  |
|  | AW - IB | -0.182 | 0.959 | 5 | -0.190 | 1 |  |
|  | AW - VB | -1.125 | 0.959 | 5 | -1.173 | 0.766 |  |
|  | GP - HZ | 0.761 | 0.959 | 5 | 0.794 | 0.922 |  |
|  | GP - IB | -0.170 | 0.959 | 5 | -0.177 | 1 |  |
|  | GP - VB | -1.113 | 0.959 | 5 | -1.161 | 0.773 |  |
|  | HZ - IB | -0.931 | 0.959 | 5 | -0.971 | 0.858 |  |
|  | HZ - VB | -1.873 | 0.959 | 5 | -1.954 | 0.398 |  |
|  | IB - VB | -0.942 | 0.959 | 5 | -0.983 | 0.853 |  |
| area_mn_high_500 | Forest - Grassland | 0.113 | 0.329 | 8 | 0.343 | 0.74 |  |
|  | AW - GP | 0.234 | 0.179 | 5 | 1.306 | 0.7 |  |
|  | AW - HZ | 0.714 | 0.179 | 5 | 3.992 | < 0.1 | . |
|  | AW - IB | 0.239 | 0.179 | 5 | 1.339 | 0.684 |  |
|  | AW - VB | -0.673 | 0.179 | 5 | -3.764 | < 0.1 | . |
|  | GP - HZ | 0.480 | 0.179 | 5 | 2.686 | 0.188 |  |
|  | GP - IB | 0.006 | 0.179 | 5 | 0.033 | 1 |  |
|  | GP - VB | -0.906 | 0.179 | 5 | -5.070 | < 0.05 | * |
|  | HZ - IB | -0.474 | 0.179 | 5 | -2.653 | 0.194 |  |
|  | HZ - VB | -1.386 | 0.179 | 5 | -7.756 | < 0.01 | ** |
|  | IB - VB | -0.912 | 0.179 | 5 | -5.103 | < 0.05 | * |
| area_mn_high_1000 | Forest - Grassland | -0.074 | 0.378 | 8 | -0.197 | 0.849 |  |
|  | AW - GP | 0.610 | 0.129 | 5 | 4.738 | < 0.05 | * |
|  | AW - HZ | 0.806 | 0.129 | 5 | 6.260 | < 0.01 | ** |
|  | AW - IB | 0.538 | 0.129 | 5 | 4.177 | < 0.05 | * |
|  | AW - VB | -0.649 | 0.129 | 5 | -5.043 | < 0.05 | * |
|  | GP - HZ | 0.196 | 0.129 | 5 | 1.522 | 0.592 |  |
|  | GP - IB | -0.072 | 0.129 | 5 | -0.560 | 0.976 |  |
|  | GP - VB | -1.259 | 0.129 | 5 | -9.781 | < 0.01 | ** |
|  | HZ - IB | -0.268 | 0.129 | 5 | -2.082 | 0.35 |  |
|  | HZ - VB | -1.455 | 0.129 | 5 | -11.303 | < 0.001 | *** |
|  | IB - VB | -1.187 | 0.129 | 5 | -9.220 | < 0.01 | ** |
| area_mn_high_1500 | Forest - Grassland | 0.058 | 0.266 | 8 | 0.218 | 0.833 |  |
|  | AW - GP | 0.361 | 0.076 | 5 | 4.759 | < 0.05 | * |
|  | AW - HZ | 0.525 | 0.076 | 5 | 6.922 | < 0.01 | ** |
|  | AW - IB | 0.372 | 0.076 | 5 | 4.900 | < 0.05 | * |
|  | AW - VB | -0.514 | 0.076 | 5 | -6.778 | < 0.01 | ** |
|  | GP - HZ | 0.164 | 0.076 | 5 | 2.163 | 0.322 |  |
|  | GP - IB | 0.011 | 0.076 | 5 | 0.141 | 1 |  |
|  | GP - VB | -0.875 | 0.076 | 5 | -11.537 | < 0.001 | *** |
|  | HZ - IB | -0.153 | 0.076 | 5 | -2.022 | 0.372 |  |
|  | HZ - VB | -1.039 | 0.076 | 5 | -13.700 | < 0.001 | *** |
|  | IB - VB | -0.886 | 0.076 | 5 | -11.678 | < 0.001 | *** |
| area_mn_high_3000 | Forest - Grassland | -0.001 | 0.084 | 8 | -0.010 | 0.992 |  |
|  | AW - GP | 0.221 | 0.012 | 5 | 17.892 | < 0.001 | *** |
|  | AW - HZ | 0.357 | 0.012 | 5 | 28.964 | < 0.001 | *** |
|  | AW - IB | 0.261 | 0.012 | 5 | 21.207 | < 0.001 | *** |
|  | AW - VB | 0.176 | 0.012 | 5 | 14.258 | < 0.001 | *** |
|  | GP - HZ | 0.137 | 0.012 | 5 | 11.072 | < 0.001 | *** |
|  | GP - IB | 0.041 | 0.012 | 5 | 3.315 | < 0.1 | . |
|  | GP - VB | -0.045 | 0.012 | 5 | -3.634 | < 0.1 | . |
|  | HZ - IB | -0.096 | 0.012 | 5 | -7.758 | < 0.01 | ** |
|  | HZ - VB | -0.181 | 0.012 | 5 | -14.706 | < 0.001 | *** |
|  | IB - VB | -0.086 | 0.012 | 5 | -6.949 | < 0.01 | ** |

**Table S7:** Summary of ANOVA tests results. These models evaluate the differences in the *high vegetation patch connectivity* (contig_mn_high) metric for site (forest vs. grassland) and location for each of the seven buffer distances. Model names are abbreviated using the metric and buffer distance.

| Model | Contrast | Estimate | SE | Df | t-ratio | P-value | Significance |
| --- | --- | --- | --- | --- | --- | --- | --- |
| contig_mn_high_25 | Forest - Grassland | 0.156 | 0.159 | 8 | 0.981 | 0.355 |  |
|  | AW - GP | 0.375 | 0.254 | 5 | 1.474 | 0.616 |  |
|  | AW - HZ | 0.306 | 0.254 | 5 | 1.203 | 0.752 |  |
|  | AW - IB | 0.000 | 0.254 | 5 | 0.000 | 1 |  |
|  | AW - VB | 0.093 | 0.254 | 5 | 0.365 | 0.995 |  |
|  | GP - HZ | -0.069 | 0.254 | 5 | -0.271 | 0.998 |  |
|  | GP - IB | -0.375 | 0.254 | 5 | -1.474 | 0.616 |  |
|  | GP - VB | -0.282 | 0.254 | 5 | -1.109 | 0.797 |  |
|  | HZ - IB | -0.306 | 0.254 | 5 | -1.203 | 0.752 |  |
|  | HZ - VB | -0.213 | 0.254 | 5 | -0.838 | 0.908 |  |
|  | IB - VB | 0.093 | 0.254 | 5 | 0.365 | 0.995 |  |
| contig_mn_high_50 | Forest - Grassland | 0.260 | 0.194 | 8 | 1.344 | 0.216 |  |
|  | AW - GP | 0.378 | 0.335 | 5 | 1.129 | 0.788 |  |
|  | AW - HZ | 0.429 | 0.335 | 5 | 1.282 | 0.712 |  |
|  | AW - IB | -0.003 | 0.335 | 5 | -0.009 | 1 |  |
|  | AW - VB | 0.065 | 0.335 | 5 | 0.196 | 1 |  |
|  | GP - HZ | 0.051 | 0.335 | 5 | 0.153 | 1 |  |
|  | GP - IB | -0.381 | 0.335 | 5 | -1.137 | 0.784 |  |
|  | GP - VB | -0.312 | 0.335 | 5 | -0.933 | 0.873 |  |
|  | HZ - IB | -0.432 | 0.335 | 5 | -1.291 | 0.708 |  |
|  | HZ - VB | -0.364 | 0.335 | 5 | -1.087 | 0.807 |  |
|  | IB - VB | 0.068 | 0.335 | 5 | 0.204 | 0.999 |  |
| contig_mn_high_250 | Forest - Grassland | 0.061 | 0.163 | 8 | 0.375 | 0.717 |  |
|  | AW - GP | 0.191 | 0.199 | 5 | 0.960 | 0.862 |  |
|  | AW - HZ | 0.351 | 0.199 | 5 | 1.763 | 0.478 |  |
|  | AW - IB | 0.055 | 0.199 | 5 | 0.276 | 0.998 |  |
|  | AW - VB | -0.197 | 0.199 | 5 | -0.988 | 0.851 |  |
|  | GP - HZ | 0.160 | 0.199 | 5 | 0.803 | 0.919 |  |
|  | GP - IB | -0.136 | 0.199 | 5 | -0.684 | 0.952 |  |
|  | GP - VB | -0.388 | 0.199 | 5 | -1.948 | 0.4 |  |
|  | HZ - IB | -0.296 | 0.199 | 5 | -1.487 | 0.609 |  |
|  | HZ - VB | -0.548 | 0.199 | 5 | -2.750 | 0.175 |  |
|  | IB - VB | -0.252 | 0.199 | 5 | -1.264 | 0.722 |  |
| contig_mn_high_500 | Forest - Grassland | -0.018 | 0.095 | 8 | -0.193 | 0.852 |  |
|  | AW - GP | 0.265 | 0.096 | 5 | 2.773 | 0.171 |  |
|  | AW - HZ | 0.218 | 0.096 | 5 | 2.286 | 0.284 |  |
|  | AW - IB | 0.086 | 0.096 | 5 | 0.897 | 0.887 |  |
|  | AW - VB | -0.031 | 0.096 | 5 | -0.326 | 0.997 |  |
|  | GP - HZ | -0.047 | 0.096 | 5 | -0.487 | 0.985 |  |
|  | GP - IB | -0.179 | 0.096 | 5 | -1.875 | 0.429 |  |
|  | GP - VB | -0.296 | 0.096 | 5 | -3.098 | 0.122 |  |
|  | HZ - IB | -0.133 | 0.096 | 5 | -1.388 | 0.659 |  |
|  | HZ - VB | -0.249 | 0.096 | 5 | -2.611 | 0.203 |  |
|  | IB - VB | -0.117 | 0.096 | 5 | -1.223 | 0.742 |  |
| contig_mn_high_1000 | Forest - Grassland | -0.040 | 0.120 | 8 | -0.330 | 0.75 |  |
|  | AW - GP | 0.397 | 0.054 | 5 | 7.352 | < 0.01 | ** |
|  | AW - HZ | 0.320 | 0.054 | 5 | 5.929 | < 0.05 | * |
|  | AW - IB | 0.242 | 0.054 | 5 | 4.487 | < 0.05 | * |
|  | AW - VB | -0.013 | 0.054 | 5 | -0.232 | 0.999 |  |
|  | GP - HZ | -0.077 | 0.054 | 5 | -1.422 | 0.642 |  |
|  | GP - IB | -0.155 | 0.054 | 5 | -2.865 | 0.156 |  |
|  | GP - VB | -0.410 | 0.054 | 5 | -7.584 | < 0.01 | ** |
|  | HZ - IB | -0.078 | 0.054 | 5 | -1.443 | 0.631 |  |
|  | HZ - VB | -0.333 | 0.054 | 5 | -6.161 | < 0.01 | ** |
|  | IB - VB | -0.255 | 0.054 | 5 | -4.718 | < 0.05 | * |
| contig_mn_high_1500 | Forest - Grassland | 0.004 | 0.108 | 8 | 0.037 | 0.971 |  |
|  | AW - GP | 0.356 | 0.013 | 5 | 26.485 | < 0.001 | *** |
|  | AW - HZ | 0.320 | 0.013 | 5 | 23.801 | < 0.001 | *** |
|  | AW - IB | 0.240 | 0.013 | 5 | 17.845 | < 0.001 | *** |
|  | AW - VB | 0.007 | 0.013 | 5 | 0.509 | 0.983 |  |
|  | GP - HZ | -0.036 | 0.013 | 5 | -2.684 | 0.188 |  |
|  | GP - IB | -0.116 | 0.013 | 5 | -8.641 | < 0.01 | ** |
|  | GP - VB | -0.349 | 0.013 | 5 | -25.976 | < 0.001 | *** |
|  | HZ - IB | -0.080 | 0.013 | 5 | -5.957 | < 0.01 | ** |
|  | HZ - VB | -0.313 | 0.013 | 5 | -23.292 | < 0.001 | *** |
|  | IB - VB | -0.233 | 0.013 | 5 | -17.335 | < 0.001 | *** |
| contig_mn_high_3000 | Forest - Grassland | 0.004 | 0.065 | 8 | 0.064 | 0.951 |  |
|  | AW - GP | 0.239 | 0.008 | 5 | 28.364 | < 0.001 | *** |
|  | AW - HZ | 0.263 | 0.008 | 5 | 31.249 | < 0.001 | *** |
|  | AW - IB | 0.152 | 0.008 | 5 | 18.025 | < 0.001 | *** |
|  | AW - VB | 0.154 | 0.008 | 5 | 18.310 | < 0.001 | *** |
|  | GP - HZ | 0.024 | 0.008 | 5 | 2.884 | 0.153 |  |
|  | GP - IB | -0.087 | 0.008 | 5 | -10.340 | < 0.001 | *** |
|  | GP - VB | -0.085 | 0.008 | 5 | -10.055 | < 0.001 | *** |
|  | HZ - IB | -0.111 | 0.008 | 5 | -13.224 | < 0.001 | *** |
|  | HZ - VB | -0.109 | 0.008 | 5 | -12.939 | < 0.001 | *** |
|  | IB - VB | 0.002 | 0.008 | 5 | 0.285 | 0.998 |  |

**Table S8:** Sampled mosquito populations at forest and grasslands sites. In total, 9,493 female mosquitoes were trapped belonging to 5 genera and identified 10 species.

| **Genus** | **Species** | **Forest** | **Grassland** |
| --- | --- | --- | --- |
| Aedes | *Aedes annulipes/cantans* | 27 | 0 |
|  | *Aedes cinereus/geminus* | 48 | 50 |
|  | *Aedes spp.* | 4 | 1 |
| Anopheles | *Anopheles maculipennis s.l.* | 9 | 8 |
|  | *Anopheles plumbeus* | 15 | 5 |
| Coquillettidia | *Coquillettidia richiardii* | 82 | 13 |
| Culex | *Culex modestus* | 2 | 6 |
|  | *Culex pipiens/torrentium* | 4,917 | 4,179 |
|  | *Culex spp.* | 1 | 1 |
|  | *Culex territans* | 0 | 1 |
| Culiseta | *Culiseta annulata* | 49 | 14 |
|  | *Culiseta morsitans* | 54 | 5 |
|  | *Culiseta spp.* | 0 | 2 |

**Table S9:** Summary of a pairwise multiple comparison between Tukey post-hoc test results in which the differences in the number of mosquitoes between site (forest vs. grassland) is evaluated per location.

| Contrast | Estimate | SE | Df | z-ratio | Pr(>Chisq) | Significance |
| --- | --- | --- | --- | --- | --- | --- |
| AW Forest - AW Grassland | -0.558 | 0.265 | 1 | -2.107 | 0.522 |  |
| GP Forest - GP Grassland | -1.113 | 0.273 | 1 | -4.083 | < 0.01 | ** |
| HZ Forest - HZ Grassland | 0.328 | 0.262 | 1 | 1.251 | 0.964 |  |
| IB Forest - IB Grassland | 0.606 | 0.261 | 1 | 2.321 | 0.375 |  |
| VB Forest - VB Grassland | 0.559 | 0.262 | 1 | 2.131 | 0.505 |  |

**Table S10:** Summary of a pairwise multiple comparison between Tukey post-hoc test results in which the differences in the number of mosquitoes is evaluated among locations.

| Contrast | Estimate | SE | Df | z-ratio | Pr(>Chisq) | Significance |
| --- | --- | --- | --- | --- | --- | --- |
| AW - GP | 0.0321 | 0.194 | 1 | 0.165 | 0.999 |  |
| AW - HZ | -0.8374 | 0.188 | 1 | -4.456 | < 0.001 | *** |
| AW - IB | -0.7896 | 0.190 | 1 | -4.151 | < 0.001 | *** |
| AW - VB | -0.7012 | 0.188 | 1 | -3.737 | < 0.001 | *** |
| GP - HZ | -0.8695 | 0.190 | 1 | -4.567 | < 0.001 | *** |
| GP - IB | -0.8217 | 0.191 | 1 | -4.303 | < 0.001 | *** |
| GP - VB | -0.7333 | 0.191 | 1 | -3.843 | < 0.001 | *** |
| HZ - IB | 0.0478 | 0.186 | 1 | 0.256 | 0.9991 |  |
| HZ - VB | 0.1362 | 0.186 | 1 | 0.733 | 0.9489 |  |
| IB - VB | 0.0884 | 0.188 | 1 | 0.469 | 0.9901 |  |

| Model | Factor | Chisq | Df | Pr(>Chisq) | Significance |
| --- | --- | --- | --- | --- | --- |
| shdi_25 | (Intercept) | 63.803 | 1 | < 0.001 | *** |
|  | site | 0.937 | 1 | 0.333 |  |
|  | shdi_25 | 3.504 | 1 | < 0.1 | . |
|  | site:shdi_25 | 1.333 | 1 | 0.248 |  |
| shdi_50 | (Intercept) | 5.172 | 1 | < 0.05 | * |
|  | site | 7.982 | 1 | < 0.01 | ** |
|  | shdi_50 | 11.159 | 1 | < 0.001 | *** |
|  | site:shdi_50 | 7.789 | 1 | < 0.01 | ** |
| shdi_250 | (Intercept) | 73.947 | 1 | < 0.001 | *** |
|  | site | 5.573 | 1 | < 0.05 | * |
|  | shdi_250 | 6.188 | 1 | < 0.05 | * |
|  | site:shdi_250 | 4.238 | 1 | < 0.05 | * |
| shdi_500 | (Intercept) | 110.203 | 1 | < 0.001 | *** |
|  | site | 2.189 | 1 | 0.139 |  |
|  | shdi_500 | 11.455 | 1 | < 0.001 | *** |
|  | site:shdi_500 | 1.606 | 1 | 0.205 |  |
| shdi_1000 | (Intercept) | 47.854 | 1 | < 0.001 | *** |
|  | site | 0.496 | 1 | 0.481 |  |
|  | shdi_1000 | 0.216 | 1 | 0.642 |  |
|  | site:shdi_1000 | 0.309 | 1 | 0.579 |  |
| shdi_1500 | (Intercept) | 19.874 | 1 | < 0.001 | *** |
|  | site | 0.832 | 1 | 0.362 |  |
|  | shdi_1500 | 0.651 | 1 | 0.42 |  |
|  | site:shdi_1500 | 0.661 | 1 | 0.416 |  |
| shdi_3000 | (Intercept) | 79.243 | 1 | < 0.001 | *** |
|  | site | 13.244 | 1 | < 0.001 | *** |
|  | shdi_3000 | 34.381 | 1 | < 0.001 | *** |
|  | site:shdi_3000 | 13.435 | 1 | < 0.001 | *** |

**Table S11:** Summary of Type III Wald chisquare tests results for various generalized linear mixed models. These models include the metric *Shannon diversity index* (shdi) and its interaction with site (forest vs. grassland). Each model corresponds to a distinct buffer distance and aims to determine if the metric and its interaction for that specific buffer distance explains the observed variance in mosquito abundance. Model names are abbreviated using the metric and buffer distance.

| Model | Factor | Chisq | Df | Pr(>Chisq) | Significance |
| --- | --- | --- | --- | --- | --- |
| shei_25 | (Intercept) | 134.363 | 1 | < 0.001 | *** |
|  | site | 8.234 | 1 | < 0.01 | ** |
|  | shei_25 | 6.114 | 1 | < 0.05 | * |
|  | site:shei_25 | 7.373 | 1 | < 0.01 | ** |
| shei_50 | (Intercept) | 15.203 | 1 | < 0.001 | *** |
|  | site | 1.673 | 1 | 0.196 |  |
|  | shei_50 | 3.367 | 1 | < 0.1 | . |
|  | site:shei_50 | 2.091 | 1 | 0.148 |  |
| shei_250 | (Intercept) | 63.525 | 1 | < 0.001 | *** |
|  | site | 7.399 | 1 | < 0.01 | ** |
|  | shei_250 | 6.845 | 1 | < 0.01 | ** |
|  | site:shei_250 | 6.473 | 1 | < 0.05 | * |
| shei_500 | (Intercept) | 107.881 | 1 | < 0.001 | *** |
|  | site | 5.746 | 1 | < 0.05 | * |
|  | shei_500 | 13.760 | 1 | < 0.001 | *** |
|  | site:shei_500 | 5.019 | 1 | < 0.05 | * |
| shei_1000 | (Intercept) | 55.309 | 1 | < 0.001 | *** |
|  | site | 0.909 | 1 | 0.34 |  |
|  | shei_1000 | 0.888 | 1 | 0.346 |  |
|  | site:shei_1000 | 0.675 | 1 | 0.411 |  |
| shei_1500 | (Intercept) | 32.235 | 1 | < 0.001 | *** |
|  | site | 1.941 | 1 | 0.164 |  |
|  | shei_1500 | 2.437 | 1 | 0.118 |  |
|  | site:shei_1500 | 1.700 | 1 | 0.192 |  |
| shei_3000 | (Intercept) | 90.528 | 1 | < 0.001 | *** |
|  | site | 17.459 | 1 | < 0.001 | *** |
|  | shei_3000 | 43.732 | 1 | < 0.001 | *** |
|  | site:shei_3000 | 17.698 | 1 | < 0.001 | *** |

**Table S12:** Summary of Type III Wald chisquare tests results for various generalized linear mixed models. These models include the metric *Shannon evenness index* (shei) and its interaction with site (forest vs. grassland). Each model corresponds to a distinct buffer distance and aims to determine if the metric and its interaction for that specific buffer distance explains the observed variance in mosquito abundance. Model names are abbreviated using the metric and buffer distance.

| Model | Factor | Chisq | Df | Pr(>Chisq) | Significance |
| --- | --- | --- | --- | --- | --- |
| per_sl_25 | (Intercept) | 27.069 | 1 | < 0.001 | *** |
|  | site | 9.844 | 1 | < 0.01 | ** |
|  | per_sl_25 | 9.478 | 1 | < 0.01 | ** |
|  | site:per_sl_25 | 9.442 | 1 | < 0.01 | ** |
| per_sl_50 | (Intercept) | 17.639 | 1 | < 0.001 | *** |
|  | site | 3.694 | 1 | < 0.1 | . |
|  | per_sl_50 | 3.376 | 1 | < 0.1 | . |
|  | site:per_sl_50 | 3.377 | 1 | < 0.1 | . |
| per_sl_250 | (Intercept) | 112.319 | 1 | < 0.001 | *** |
|  | site | 25.441 | 1 | < 0.001 | *** |
|  | per_sl_250 | 34.965 | 1 | < 0.001 | *** |
|  | site:per_sl_250 | 21.994 | 1 | < 0.001 | *** |
| per_sl_500 | (Intercept) | 121.215 | 1 | < 0.001 | *** |
|  | site | 38.776 | 1 | < 0.001 | *** |
|  | per_sl_500 | 56.003 | 1 | < 0.001 | *** |
|  | site:per_sl_500 | 37.268 | 1 | < 0.001 | *** |
| per_sl_1000 | (Intercept) | 82.499 | 1 | < 0.001 | *** |
|  | site | 23.328 | 1 | < 0.001 | *** |
|  | per_sl_1000 | 37.870 | 1 | < 0.001 | *** |
|  | site:per_sl_1000 | 23.057 | 1 | < 0.001 | *** |
| per_sl_1500 | (Intercept) | 72.761 | 1 | < 0.001 | *** |
|  | site | 16.777 | 1 | < 0.001 | *** |
|  | per_sl_1500 | 35.079 | 1 | < 0.001 | *** |
|  | site:per_sl_1500 | 16.591 | 1 | < 0.001 | *** |
| per_sl_3000 | (Intercept) | 87.950 | 1 | < 0.001 | *** |
|  | site | 14.347 | 1 | < 0.001 | *** |
|  | per_sl_3000 | 36.631 | 1 | < 0.001 | *** |
|  | site:per_sl_3000 | 14.226 | 1 | < 0.001 | *** |

**Table S13:** Summary of Type III Wald chisquare tests results for various generalized linear mixed models. These models include the metric *landscape suitability score* (per_sl) and its interaction with site (forest vs. grassland). Each model corresponds to a distinct buffer distance and aims to determine if the metric and its interaction for that specific buffer distance explains the observed variance in mosquito abundance. Model names are abbreviated using the metric and buffer distance.

| Model | Factor | Chisq | Df | Pr(>Chisq) | Significance |
| --- | --- | --- | --- | --- | --- |
| pland_high_25 | (Intercept) | 9.899 | 1 | < 0.01 | ** |
|  | site | 3.198 | 1 | < 0.1 | . |
|  | pland_high_25 | 3.001 | 1 | < 0.1 | . |
|  | site:pland_high_25 | 3.071 | 1 | < 0.1 | . |
| pland_high_50 | (Intercept) | 11.596 | 1 | < 0.001 | *** |
|  | site | 0.446 | 1 | 0.504 |  |
|  | pland_high_50 | 0.301 | 1 | 0.583 |  |
|  | site:pland_high_50 | 0.338 | 1 | 0.561 |  |
| pland_high_250 | (Intercept) | 118.493 | 1 | < 0.001 | *** |
|  | site | 12.186 | 1 | < 0.001 | *** |
|  | pland_high_250 | 12.248 | 1 | < 0.001 | *** |
|  | site:pland_high_250 | 9.710 | 1 | < 0.01 | ** |
| pland_high_500 | (Intercept) | 224.363 | 1 | < 0.001 | *** |
|  | site | 31.135 | 1 | < 0.001 | *** |
|  | pland_high_500 | 50.861 | 1 | < 0.001 | *** |
|  | site:pland_high_500 | 30.911 | 1 | < 0.001 | *** |
| pland_high_1000 | (Intercept) | 237.638 | 1 | < 0.001 | *** |
|  | site | 18.833 | 1 | < 0.001 | *** |
|  | pland_high_1000 | 45.258 | 1 | < 0.001 | *** |
|  | site:pland_high_1000 | 21.491 | 1 | < 0.001 | *** |
| pland_high_1500 | (Intercept) | 256.474 | 1 | < 0.001 | *** |
|  | site | 21.343 | 1 | < 0.001 | *** |
|  | pland_high_1500 | 50.118 | 1 | < 0.001 | *** |
|  | site:pland_high_1500 | 25.472 | 1 | < 0.001 | *** |
| pland_high_3000 | (Intercept) | 241.610 | 1 | < 0.001 | *** |
|  | site | 14.157 | 1 | < 0.001 | *** |
|  | pland_high_3000 | 31.480 | 1 | < 0.001 | *** |
|  | site:pland_high_3000 | 15.210 | 1 | < 0.001 | *** |

**Table S14:** Summary of Type III Wald chisquare tests results for various generalized linear mixed models. These models include the metric *high vegetation cover* (pland_high) and its interaction with site (forest vs. grassland). Each model corresponds to a distinct buffer distance and aims to determine if the metric and its interaction for that specific buffer distance explains the observed variance in mosquito abundance. Model names are abbreviated using the metric and buffer distance.

| Model | Factor | Chisq | Df | Pr(>Chisq) | Significance |
| --- | --- | --- | --- | --- | --- |
| area_mn_high_25 | (Intercept) | 13.674 | 1 | < 0.001 | *** |
|  | site | 5.675 | 1 | < 0.05 | * |
|  | area_mn_high_25 | 5.609 | 1 | < 0.05 | * |
|  | site:area_mn_high_25 | 5.577 | 1 | < 0.05 | * |
| area_mn_high_50 | (Intercept) | 10.952 | 1 | < 0.001 | *** |
|  | site | 0.143 | 1 | 0.706 |  |
|  | area_mn_high_50 | 0.117 | 1 | 0.732 |  |
|  | site:area_mn_high_50 | 0.077 | 1 | 0.782 |  |
| area_mn_high_250 | (Intercept) | 173.575 | 1 | < 0.001 | *** |
|  | site | 0.076 | 1 | 0.783 |  |
|  | area_mn_high_250 | 0.667 | 1 | 0.414 |  |
|  | site:area_mn_high_250 | 0.790 | 1 | 0.374 |  |
| area_mn_high_500 | (Intercept) | 175.504 | 1 | < 0.001 | *** |
|  | site | 0.238 | 1 | 0.625 |  |
|  | area_mn_high_500 | 0.917 | 1 | 0.338 |  |
|  | site:area_mn_high_500 | 1.452 | 1 | 0.228 |  |
| area_mn_high_1000 | (Intercept) | 211.872 | 1 | < 0.001 | *** |
|  | site | 0.105 | 1 | 0.746 |  |
|  | area_mn_high_1000 | 0.012 | 1 | 0.913 |  |
|  | site:area_mn_high_1000 | 0.165 | 1 | 0.684 |  |
| area_mn_high_1500 | (Intercept) | 213.396 | 1 | < 0.001 | *** |
|  | site | 0.004 | 1 | 0.948 |  |
|  | area_mn_high_1500 | 0.149 | 1 | 0.699 |  |
|  | site:area_mn_high_1500 | 0.779 | 1 | 0.377 |  |
| area_mn_high_3000 | (Intercept) | 164.958 | 1 | < 0.001 | *** |
|  | site | 4.921 | 1 | < 0.05 | * |
|  | area_mn_high_3000 | 15.402 | 1 | < 0.001 | *** |
|  | site:area_mn_high_3000 | 4.404 | 1 | < 0.05 | * |

**Table S15:** Summary of Type III Wald chisquare tests results for various generalized linear mixed models. These models include the metric *high vegetation patch area* (area_mn_high) and its interaction with site (forest vs. grassland). Each model corresponds to a distinct buffer distance and aims to determine if the metric and its interaction for that specific buffer distance explains the observed variance in mosquito abundance. Model names are abbreviated using the metric and buffer distance.

| Model | Factor | Chisq | Df | Pr(>Chisq) | Significance |
| --- | --- | --- | --- | --- | --- |
| contig_mn_high_25 | (Intercept) | 8.180 | 1 | < 0.01 | ** |
|  | site | 3.427 | 1 | < 0.1 | . |
|  | contig_mn_high_25 | 3.504 | 1 | < 0.1 | . |
|  | site:contig_mn_high_25 | 3.260 | 1 | < 0.1 | . |
| contig_mn_high_50 | (Intercept) | 5.805 | 1 | < 0.05 | * |
|  | site | 2.068 | 1 | 0.15 |  |
|  | contig_mn_high_50 | 2.093 | 1 | 0.148 |  |
|  | site:contig_mn_high_50 | 1.921 | 1 | 0.166 |  |
| contig_mn_high_250 | (Intercept) | 202.190 | 1 | < 0.001 | *** |
|  | site | 0.010 | 1 | 0.921 |  |
|  | contig_mn_high_250 | 0.103 | 1 | 0.748 |  |
|  | site:contig_mn_high_250 | 0.302 | 1 | 0.583 |  |
| contig_mn_high_500 | (Intercept) | 125.706 | 1 | < 0.001 | *** |
|  | site | 0.054 | 1 | 0.816 |  |
|  | contig_mn_high_500 | 0.020 | 1 | 0.887 |  |
|  | site:contig_mn_high_500 | 0.455 | 1 | 0.5 |  |
| contig_mn_high_1000 | (Intercept) | 161.472 | 1 | < 0.001 | *** |
|  | site | 0.013 | 1 | 0.91 |  |
|  | contig_mn_high_1000 | 0.139 | 1 | 0.709 |  |
|  | site:contig_mn_high_1000 | 0.117 | 1 | 0.733 |  |
| contig_mn_high_1500 | (Intercept) | 133.056 | 1 | < 0.001 | *** |
|  | site | 0.015 | 1 | 0.903 |  |
|  | contig_mn_high_1500 | 0.040 | 1 | 0.841 |  |
|  | site:contig_mn_high_1500 | 0.331 | 1 | 0.565 |  |
| contig_mn_high_3000 | (Intercept) | 87.236 | 1 | < 0.001 | *** |
|  | site | 0.653 | 1 | 0.419 |  |
|  | contig_mn_high_3000 | 3.539 | 1 | < 0.1 | . |
|  | site:contig_mn_high_3000 | 0.341 | 1 | 0.559 |  |

**Table S16:** Summary of Type III Wald chisquare tests results for various generalized linear mixed models. These models include the metric *high vegetation patch connectivity* (contig_mn_high) and its interaction with site (forest vs. grassland). Each model corresponds to a distinct buffer distance and aims to determine if the metric and its interaction for that specific buffer distance explains the observed variance in mosquito abundance. Model names are abbreviated using the metric and buffer distance.

**Table S17:** Sampled bird population at forest and grasslands sites. In total, 566 birds were observed belonging to 45 different species.

| **Family** | **Scientific name** | **English common name** | **Forest** | **Grassland** |
| --- | --- | --- | --- | --- |
| Accipitridae | *Buteo buteo* | Common Buzzard | 1 | 1 |
| Acrocephalidae | *Acrocephalus palustris* | Marsh Warbler | 1 | 0 |
|  | *Acrocephalus schoenobaenus* | Sedge Warbler | 5 | 17 |
|  | *Acrocephalus scirpaceus* | Eurasian Reed Warbler | 7 | 15 |
|  | *Hippolais icterina* | Icterine Warbler | 3 | 0 |
| Ardeidae | *Alopochen aegyptiaca* | Egyptian Goose | 0 | 3 |
|  | *Anas platyrhynchos* | Mallard | 1 | 4 |
|  | *Anser anser* | Greylag Goose | 0 | 3 |
|  | *Ardea cinerea* | Blue Heron | 0 | 1 |
| Certhiidae | *Certhia brachydactyla* | Short-toed Treecreeper | 1 | 0 |
| Charadriidae | *Vanellus vanellus* | Northern Lapwing | 1 | 0 |
| Columbidae | *Columba palumbus* | Common Wood Pigeon | 2 | 5 |
| Corvidae | *Corvus corone* | Carrion Crow | 2 | 1 |
|  | *Garrulus glandarius* | Jay | 2 | 0 |
| Emberizidae | *Emberiza schoeniclus* | Reed Bunting | 0 | 21 |
| Falconidae | *Falco tinnunculus* | Common Kestrel | 0 | 3 |
| Fringillidae | *Carduelis carduelis* | European Goldfinch | 1 | 8 |
|  | *Fringilla coelebs* | Common Chaffinch | 41 | 3 |
| Hirundinidae | *Delichon urbicum* | House Martin | 0 | 4 |
|  | *Hirundo rustica* | Barn Swallow | 0 | 11 |
| Laridae | *Sterna hirundo* | Common Tern | 0 | 2 |
| Locustellidae | *Locustella luscinioides* | Savis Warbler | 0 | 4 |
| Motacillidae | *Motacilla alba* | White Wagtail | 0 | 1 |
| Muscicapidae | *Erithacus rubecula* | European Robin | 18 | 0 |
|  | *Saxicola torquatus* | European Stonechat | 0 | 2 |
| Paridae | *Cyanistes caeruleus* | Eurasian Blue Tit | 21 | 8 |
|  | *Parus major* | Great Tit | 29 | 5 |
|  | *Poecile palustris* | Marsh Tit | 5 | 0 |
| Phasianidae | *Phasianus colchicus* | Common Pheasant | 0 | 1 |
| Phylloscopidae | *Phylloscopus collybita* | Common Chiffchaff | 75 | 8 |
|  | *Phylloscopus trochilus* | Willow Warbler | 7 | 0 |
| Picidae | *Dendrocopos major* | Great Spotted Woodpecker | 5 | 1 |
|  | *Picus viridis* | Eurasian Green Woodpecker | 1 | 0 |
| Prunellidae | *Prunella modularis* | Dunnock | 1 | 3 |
| Rallidae | *Fulica atra* | Eurasian Coot | 0 | 5 |
|  | *Rallus aquaticus* | Water Rail | 0 | 1 |
| Regulidae | *Regulus regulus* | Goldcrest | 1 | 0 |
| Sturnidae | *Sturnus vulgaris* | Common Starling | 0 | 3 |
| Sylviidae | *Sylvia atricapilla* | Eurasian Blackcap | 42 | 0 |
|  | *Sylvia borin* | Garden Warbler | 3 | 0 |
|  | *Sylvia communis* | Common Whitethroat | 2 | 0 |
|  | *Sylvia curruca* | Lesser Whitethroat | 3 | 0 |
| Troglodytidae | *Troglodytes troglodytes* | Eurasian Wren | 45 | 1 |
| Turdidae | *Turdus merula* | Common Blackbird | 67 | 13 |
|  | *Turdus philomelos* | Song Thrush | 15 | 0 |
